# Phytotoxic Metabolites Isolated from *Aspergillus* sp., an Endophytic Fungus of *Crassula arborescens*

**DOI:** 10.3390/molecules27227710

**Published:** 2022-11-09

**Authors:** Jingjing Ma, Chunhua Lu, Yajie Tang, Yuemao Shen

**Affiliations:** 1 Key Laboratory of Chemical Biology (Ministry of Education), School of Pharmaceutical Sciences, Cheeloo College of Medicine, Shandong University, Jinan 250012, China; 2 State Key Laboratory of Microbial Technology, Shandong University, Qingdao 266237, China

**Keywords:** *Crassula arborescens*, endophytic fungus, *Aspergillus* sp., phytotoxic activity

## Abstract

*Aspergillus* sp., an endophytic fungus isolated from *Crassula arborescens*, displayed potent inhibitory activity against the seed germination of *Arabidopsis thaliana*. The bioactivity-guided fractionation of the culture extract of *Aspergillus* sp. MJ01 led to the isolation of nine compounds, including one previously undescribed furanone, namely aspertamarinoic acid (**1**), and eight known compounds, (−)-dihydrocanadensolide (**2**), kojic acid (**3**), citreoisocoumarin (**4**), astellolide A (**5**), astellolide B (**6**), astellolide G (**7**), *cyclo*-N-methylphenylalanyltryptophenyl (**8**) and (−)-ditryptophenaline (**9**). In the evaluation of the phytotoxic activities of compounds **1**–**9**, the results suggested that **1** and **5** showed significant inhibitory activity on the seed germination of *A. thaliana*. This is the first report to disclose the phytotoxic activity of these compounds.

## 1. Introduction

According to statistics from the Food and Agriculture Organization (FAO) of the United Nations, there are more than 8000 weeds endangering the growth of crops all over the world; weed damage is one of the main reasons for crop yield reduction [1,2,3,4]. However, the excessive use of chemical herbicides has also caused a series of problems, such as serious environmental pollution, harm to human health and the emergence of herbicide resistant weed plants. Compared with chemical herbicides containing chlorine halogens, bio-herbicides are more diverse, more easily degraded in the environment and more environmentally friendly. In recent years, with the enhancement of human environmental awareness and the need of sustainable agricultural development, it has become an inevitable trend of herbicide development to find safer, more effective and more selective bio-herbicides [5]. In the natural environment, many plants and microorganisms can produce some phytotoxic natural products to inhibit the growth of other plants around them, which is one of the primary causes why they can dominate the population competition [6]. Therefore, the development of phytotoxic natural products as bio-herbicides or lead compounds for agricultural production has become the current research hotspot [7].

*Crassula arborescens*, a succulent subshrub of genus *Crassula* L. in the Crassulaceae family, is native to South Africa and has been introduced and cultivated in China. It has been proven that some phenolic acids isolated from *C. arborescens* had physiological activities, such as anti-microbial, anti-oxidative and anti-inflammatory activities [8]. Through long-term observation, we found that there are fungal-like substances on the naturally falling leaves of *C. arborescens*. Therefore, we isolated endophytic fungi from the healthy leaves of *C. arborescens* and tested their phytotoxic activity. In this study, *Aspergillus* sp. MJ01 was isolated as one of the dominant endophytic fungi in *C. arborescens*, and its fermentation extract exhibited a strong inhibitory effect on the seed germination of *Arabidopsis thaliana*. Therefore, the bioactivity-guided fractionation of the phytotoxic metabolites from fermentation extract of *Aspergillus* sp. MJ01 was carried out, nine compounds were identified, and their inhibitory effects on the seed germination of *A. thaliana* were evaluated. This study provides potential bio-herbicides or lead compounds for weed control in agriculture.

## 2. Results

### 2.1. Identification of Strain MJ01

Strain MJ01 was isolated from healthy leaves of *C. arborescens*, and its secondary metabolites were found to have remarkable inhibitory activity on the seed germination of *A. thaliana*. The colony of strain MJ01 was flocculent, green in color, and the conidia were spherical, green, with spinules (Figure S25). On the basis of the colony, microscopic and mycelium characteristics and compared with the strains of *Aspergillus* in the literature [9], strain MJ01 was identified as *Aspergillus* sp.

**Figure 1:**
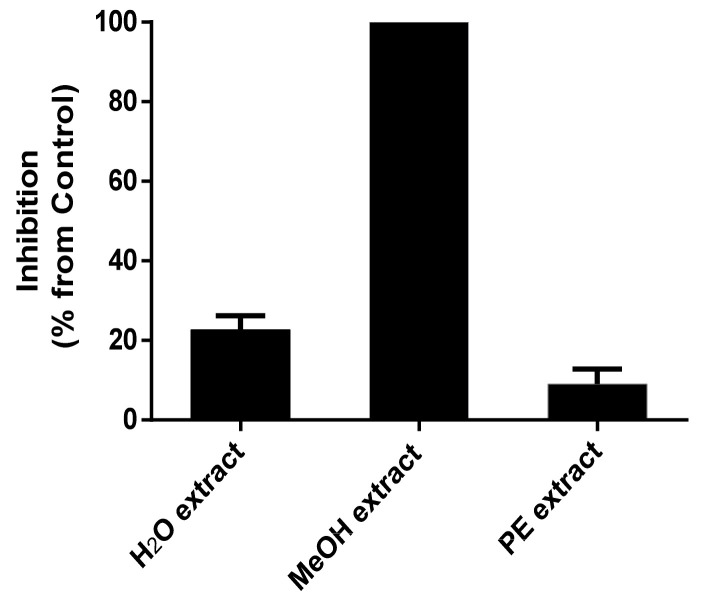
Inhibitory activity of H_2_O, MeOH and PE extracts on the seed germination of *A. thaliana* (at 50 μg for 72 h).

The inhibitory effects of water, methanol (MeOH) and petroleum ether (PE) extracts of strain MJ01 were evaluated on the seed germination of *A. thaliana*. Results indicated that the MeOH extract displayed the most potent phytotoxic natural product on the seed germination of *A. thaliana*, which caused complete inhibition of the seed germination at the dosage of 50 μg (Figure 1). Therefore, the MeOH extract was further separated by bioassay-guided column chromatography (CC) over silica gel, Sephadex LH-20 and RP-18 (MPLC).

### 2.2. Isolation, Purification and Structure Elucidation

The active MeOH extract of the cultures of strain MJ01 was subjected to CC on silica gel to provide 14 fractions (Fr. A–Fr. N). Among these fractions, nine compounds were purified by repeated CC. Their structures were identified to be aspertamarinoic acid (**1**), (−)-dihydrocanadensolide (**2**) [10], kojic acid (**3**) [11], citreoisocoumarin (**4**) [12,13], astellolide A (**5**) [14], astellolide B (**6**) [15], astellolide G (**7**) [16], *cyclo*-N-methylphenylalanyltryptophenyl (**8**) [17] and (−)-ditryptophenaline (**9**) [18] (Figure 2) on the basis of their HRESIMS, 1D and 2D NMR spectra (see Figures S1–S24) and compared with those reported in the previous literature.

Compound **1** was isolated as a colorless gum. The molecular formula was determined to be C_11_H_16_O_5_, deduced by HRESIMS [M − H]^−^ ion peak at *m/z* 227.0923. The ^1^H NMR spectrum data of **1** displayed the signals of two oxygenated methine protons at δ_H_ 5.09 (quint, *J* = 1.8 Hz, H-6a) and 4.14 (ddd, *J* = 1.4, 5.6, 7.4 Hz, H-6), two methyl groups at δ_H_ 2.13 (d, *J* = 2.0 Hz, H_3_-3′) and 0.96 (t, *J* = 7.1 Hz, H_3_-10) and three methylene groups at δ_H_ 1.70 (m, 2H, H_2_-7), 1.42 (m, 2H, H_2_-9), 1.53 and 1.43 (each m, 1H, H_ab_-8). The ^13^C NMR data showed the signals of two carbonyl carbons at δ_C_ 176.1 (C-2) and 166.4 (C-4), two unsaturated quaternary carbons at δ_C_ 150.8 (C-3a) and 136.5 (C-3), two oxygenated methine carbons at δ_C_ 85.7 (C-6a) and 70.6 (C-6), three methylene carbons at δ_C_ 35.0 (C-7), 29.5 (C-8) and 23.7 (C-9) and two methyl carbons at δ_C_ 14.5 (C-10) and 10.7 (C-3′). The presence of the *n*-butyl group was determined by the HMBC correlations of H_2_-7 with C-8 and C-9, H_3_-10 with C-8 and C-9 as well as the ^1^H-^1^H COSY correlations of H_ab_-8/H_2_-7 and H_2_-9, H_2_-9/H_3_-10. The HMBC correlation between H-6 with C-7 and C-8 as well as the ^1^H-^1^H COSY correlations of H_2_-7/H-6 manifested the presence of 1-substituted-1-hydroxypentyl unit. Additionally, the HMBC correlations between H_3_-3′ with C-2, C-3 and C-3a, H-6a with C-3a indicated the presence of α, β-unsaturated γ-lactone ring with a methyl located at the C-3. The ^1^H-^1^H COSY correlation of H-6/H-6a suggested the 1-hydroxypentyl group was substituted at C-6a. The NOESY correlations of H_2_-8b/H-6 and H_2_-8b/H-6a indicated these protons to be co-facial. According to the 1D and 2D NMR data, the planar structure of **1** was similar to aspertamarinolide A [19] whose carboxyl group located at C-4 was esterified. Additionally, the experimental ECD spectrum of **1** (Figure S1) was basically the same as that of aspertamarinolide A, and they had the same sign of the specific rotation (**1**: ${\left[\alpha \right]}_{D}^{20}\;$ − 316.1; aspertamarinolide A: ${\left[\alpha \right]}_{D}^{25}\;$ − 18.7), which suggested that these two compounds had the same absolute configurations, and the absolute configuration of **1** was assigned as 6*R* and 6a*S*. Based on the above analysis, **1** was established as aspertamarinoic acid.

**Figure 3:**
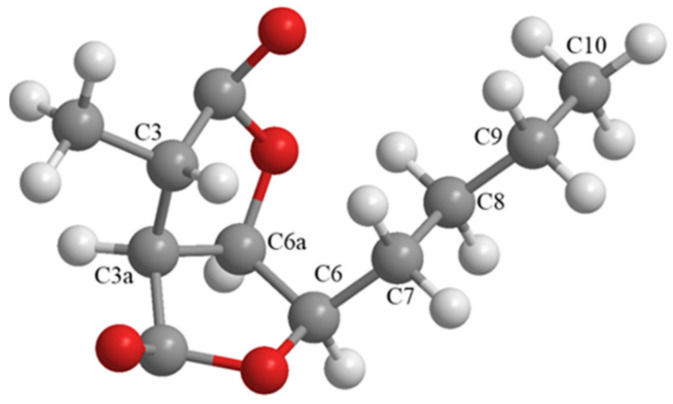
Oak ridge thermal ellipsoid plot (ORTEP) diagram for crystal structure of (−)-dihydrocanadensolide (**2**).

Compound **2** was isolated as a colorless needle crystal after slow solvent evaporation. The ^1^H and ^13^C NMR data of **2** and the HMBC correlations of H_3_-3′ with C-3, C-3a and C-2, H-6a with C-6 and C-4 as well as the ^1^H-^1^H COSY correlation of H-6a/H-6 and H-3a indicated that **2** had the same planar structure as dihydrocanadensolide [10,20,21]. Because of the presence of four chiral centers, the absolute configuration of dihydrocanadensolide has been controversial. For example, Lars Kattner reported that the absolute configuration of dihydrocanadensolide was assigned as 3*S*, 3a*S*, 6*R*, 6a*R* with ${\left[\alpha \right]}_{D}^{20}$ − 31 (c 0.26, MeOH) in 1990 [10]. However, T. Gopinath reported that the absolute configuration of (+)-dihydrocanadensolide was also assigned as 3*S*, 3a*S*, 6*R*, 6a*R* but with ${\left[\alpha \right]}_{D\;}$ + 10.12 (c 0.45, CHCl_3_) in 2003 [20]. They had the same absolute configuration but their specific rotation was opposite in these two studies. In this study, we identified the absolute stereochemistry of **2** as 3*S*, 3a*S*, 6*R*, 6a*R* by single-crystal X-ray diffraction (CCDC No. 2213294) (Figure 3). Comparison of the data showed that **2** was the enantiomer of (+)-dihydrocanadensolide whose absolute configuration was assigned as 3*R*, 3a*R*, 6*S*, 6a*S* with opposite specific rotation (**2**: ${\left[\alpha \right]}_{D}^{20}\;$ − 24.0 (c 0.10, MeOH); (+)-dihydrocanadensolide: ${\left[\alpha \right]}_{D}^{23}\;$ + 30.0) [21]. Therefore, the absolute configuration of (+)-dihydrocanadensolide determined by T. Gopinath might not be correct and we identified **2** as (−)-dihydrocanadensolide.

### 2.3. Bioactivity of Purified Compounds

The germination-inhibition assays of compounds **1**–**9** and the minimum inhibitory concentrations (MICs) of active compounds were tested on the seed of *A. thaliana*.

**Figure 4:**
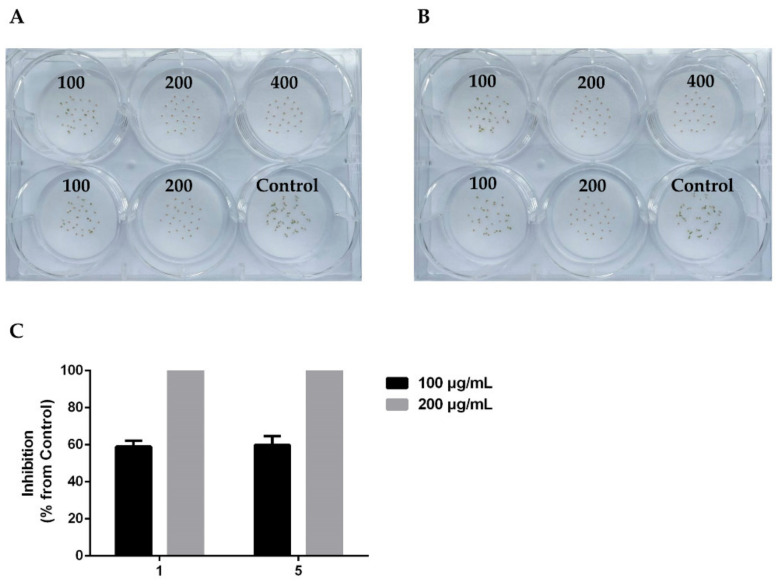
Effect of compounds **1** (**A**) and **5** (**B**) on *A. thaliana* seed germination at different concentrations (μg/mL) after 72 h of treatment. (**C**) The inhibitory rate of **1** and **5** at 100 μg/mL and 200 μg/mL, respectively.

As shown in Table 1, compounds **1** and **5** exhibited significant inhibitory activity, completely inhibiting the seed germination of *A. thaliana* at a concentration of 200 μg/mL (Figure 4). Compounds **3** and **8** with moderate activity were able to completely inhibit the seed germination of *A. thaliana* at a concentration of 400 μg/mL. Compounds **2** and **4** displayed weak inhibitory activity against the seed germination at a concentration of 800 μg/mL. In addition, other compounds were found to have no activity.

## 3. Discussion

Endophytes are microorganisms that live in the tissues of healthy plants without causing visible disease symptoms [22]. Investigations have shown that all higher plants are hosts of one or more endophytes [23]. Endophytes play an important role in protecting plant growth, and they enhance plant resistance to biotic and abiotic stresses [24]. Recently, endophytes have attracted more attention because they can be used as one of the sources of novel natural products with biological activity [25,26,27,28,29,30]. The kinds of phytotoxic natural products with the potential to be developed as bio-herbicide have been identified from endophytic fungi, such as agropyrenol from *Ascochyta agropyrina* [31], chaetoglobosin A from *Chaetomium globosum* [32] and cytochalasin E from *Xylaria* sp. [33].

Previous research has indicated that several α-pyrones had phytotoxic activities, such as 6-Pentyl-α-pyrone from *Trichoderma harzianum* [34], alternapyrones E and F from *Parastagonospora nodorum* [35] and alterpyrone A from *Alternaria brassicicola* [36]. The star molecule that has been regarded as the research hotspot was germicidin, an 6-(2-butyl)-3-ethyl-4-hydroxy-2-pyrone isolated from *Streptomyces viridochromogenes*, which could inhibit its own arthrospores germination at a concentration as low as 40 pg/mL and reduce the apical tip growth of *Lepidium sativum* at a concentration of 100 μg/mL [37]. Compared with germicidin, citreoisocoumarin (**4**), a benzo-α-pyrone, exhibited relatively weak phytotoxic activity, which further confirmed that 2-butyl chain as well as the enolic OH-group were obviously essential for inhibitory.

In this study, we found that a 2-hydroxymethyl-5-hydroxy-γ-pyrone (kojic acid (**3**), could completely inhibit the seed germination of *A. thaliana* at a concentration of 400 μg/mL. Meanwhile, aspertamarinoic acid (**1**), a 3-furancarboxylic acid, was able to completely inhibit the seed germination of *A. thaliana* at a concentration of 200 μg/mL. Compared with aspertamarinoic acid (**1**), (−)-dihydrocanadensolide (**2**) exhibited relatively weak phytotoxic activity, so we speculated that the double bond of furan ring and the ring opening of lactone may be essential factors affecting its inhibitory activities. Compounds **5**–**7** are drimane-type sesquiterpenoids with a furanone group without cytotoxic activity isolated from *Aspergillus* sp. [16]. Compared with astellolides B (**6**) and G (**7**), astellolide A (**5**) exhibited obvious phytotoxic activity, which may be related to the benzene ring without the phenol hydroxyl group. (−)-ditryptophenaline (**9**) is the dimeric product of *cyclo*-N-methylphenylalanyltryptophenyl (**8**). Interestingly, **8** exhibited more significant phytotoxic activity than its dimer against the seed germination of *A. thaliana*.

## 4. Materials and Methods

### 4.1. General Experimental Procedures

NMR spectra were recorded on DRX-400 and DRX-600 MHz NMR spectrometer (Bruker, Billerica, MA, USA) with tetramethylsilane (TMS) as an internal standard. HRESIMS were carried out on a LTQ-Orbitrap XL (Thermo Finnigan, San Jose, CA, USA). Specific rotation was obtained on an IP-digi300 polarimeter (InsMark, Shanghai, China) at 20 °C. SC-XRD data were collected on a Bruker D8 venture diffractometer (Bruker, Billerica, MA, USA). Silica gel (200–300 mesh; Qingdao Haiyang Chemical Co., Ltd., Qingdao, China), LiChroprep RP-18 (40–63 mm; Darmstadt, Germany) and Sephadex LH-20 (25–100 μm; Pharmacia Biotek, Copenhagen, Denmark) were used for column chromatography. Thin-layer chromatography (TLC) was carried out with glass precoated silica gel GF_254_ plates (Qingdao Haiyang Chemical Co., Ltd.). Compounds were visualized under UV light and by spraying with phosphomolybdic acid followed by heating. All solvents were analytical grade.

### 4.2. Fungus Isolation

The endophytic fungus strain MJ01 was isolated from the surface-sterilized healthy leaves of *C. arborescens*, a succulent subshrub growing in Jinan, Shandong province, China.

The healthy leaves of *C. arborescens* were first washed with running tap water, then sterilized by soaking in 75% ethanol for 60 s, and finally rinsed with sterile distilled water. The surface-sterilized leaves were placed on the sterilized filter paper and cut into 5 × 5 mm pieces. Afterwards, three pieces were placed in the 9 cm petri dishes containing potato dextrose agar (PDA) media. Plates were incubated at 28 °C and observed daily. The individual hypha tips of the emerging colonies were re-inoculated in fresh PDA plates until pure cultures were obtained and preserved on PDA slants at 4 °C [38].

On the basis of the colony, microscopic and mycelium characteristics, strain MJ01 was identified as *Aspergillus* sp. and has been deposited at the School of Pharmaceutical Science, Shandong University.

### 4.3. Fermentation, Extraction, Bioassay-Guided Fractionation and Purification of Compounds

*Aspergillus* sp. MJ01 was cultured for 15 d on 9 cm petri dishes with 20 mL PDA medium at 28 °C. The culture agar (10 L) was chopped, diced, and extracted three times with EtOAc-MeOH-AcOH (80: 15: 5 *v*/*v*/*v*) at room temperature overnight. The organic solution was collected by filtration and the combined filtrates were concentrated to remove the organic solvents. Then, the extract was partitioned between H_2_O and EtOAc until the EtOAc layer was colorless. Afterwards, the EtOAc solution was dried over anhydrous Na_2_SO_4_, and the solvent was removed under vacuum. The EtOAc extract was partitioned with PE and 95% aqueous MeOH until the PE layer was colorless. The MeOH solution was concentrated under vacuum to obtain MeOH extract.

PE and MeOH extracts were prepared for the seed germination bioassay of *A. thaliana*. The MeOH extract with strong inhibitory effect was further isolated and purified. The MeOH extract (~9.9 g) was submitted to MPLC over RP-18 column (120 g) eluted with 30%, 50%, 70% and 100% MeOH to give 42 fractions (220 mL each). Fourteen fractions (Fr. A–Fr. N) were pooled according to TLC detection results. Fr. G–Fr. L showed obvious activity against the seed germination of *A. thaliana* and were selected for further isolation and purification. Fr. G (275 mg) was subjected to CC over silica gel (20 g) eluted with gradient CH_2_Cl_2_-MeOH (50:1) to yield **3** (5 mg). Fr. F (40 mg) was isolated by CC over silica gel (4 g) eluted with gradient PE-EtOAc (5:1, 4:1, 1:1) and Sephadex LH-20 column (10 g) eluted with MeOH to give **4** (1 mg). Fr. H (358 mg) was chromatographed on Sephadex LH-20 column (120 g) eluted with MeOH to provide Fr. H1–Fr. H5. Fr. H1 (26 mg) was purified by CC over silica gel (3 g) eluted with gradient PE-EtOAc (5:1, 3:1, 1:1) to afford **7** (4 mg). Fr. H3 (24 mg) was applied to CC over silica gel (3 g) eluted with gradient PE-EtOAc (15:1, 10:1, 5:1, 1:1) to yield **8** (10 mg). Fr. J (170 mg) was isolated by CC on silica gel (15 g) eluted with gradient PE-EtOAc (10:1, 6:1, 4:1) to obtain **2** (4 mg) and **6** (5 mg). Fr. K (605 mg) was submitted to CC on Sephadex LH-20 (120 g) eluted with MeOH to yield Fr. K1–Fr. K3. Fr. K1 (66.8 mg) was applied to CC over silica gel (5 g) eluted with gradient PE-EtOAc (6:1, 4:1, 2:1) to give **9** (15 mg). Fr. K3 (50 mg) was chromatographed on silica gel column (3 g) eluted with gradient CH_2_Cl_2_-MeOH (100:1, 50:1) to afford **1** (3 mg) and **5** (4 mg).

#### 4.3.1. Aspertamarinoic Acid (**1**)

^1^H NMR (CD_3_OD, 600 MHz) δ 5.09 (1H, m, *J* = 1.8 Hz, H-6a), 4.14 (1H, ddd, *J* = 1.4, 5.6, 7.4 Hz, H-6), 2.13 (3H, d, *J* = 2.0 Hz, H_3_-3′), 1.70 (2H, m, H_2_-7), 1.53 (1H, m, H_2_-8a), 1.43 (1H, m, H_2_-8b), 1.42 (2H, m, H_2_-9), 0.96 (3H, t, *J* = 7.1 Hz, H_3_-10); ^13^C NMR (CD_3_OD, 150 MHz) δ 176.1 (C-2), 166.4 (C-4), 150.8 (C-3a), 136.5 (C-3), 85.7 (C-6a), 70.6 (C-6), 35.0 (C-7), 29.5 (C-8), 23.7 (C-9), 14.5 (C-10), 10.7 (Me).$\;{\left[\alpha \right]}_{D}^{20}$ − 316.0 (c 0.10, MeOH). (−)-HRESIMS *m*/*z* 227.0923 [M − H]^−^ (calcd for C_11_H_15_O_5_^−^, 227.0925).

#### 4.3.2. (−)-Dihydrocanadensolide (**2**)

^1^H NMR (CDCl_3_, 600 MHz) δ 5.13 (1H, dd, *J* = 3.9, 6.1 Hz, H-6a), 4.57 (1H, ddd, *J* = 3.9, 6.4, 8.0 Hz, H-6), 3.16 (1H, dd, *J* = 1.4, 6.1 Hz, H-3a), 3.09 (1H, qd, *J* = 1.4, 7.7 Hz, H-3), 1.95 (1H, m, H_2_-7a), 1.86 (1H, m, H_2_-7b), 1.51 (2H, m, H_2_-8), 1.46 (3H, d, *J* = 7.7 Hz, Me), 1.43 (2H, m, H_2_-9), 0.95 (3H, t, *J* = 7.3 Hz, H_3_-10); ^13^C NMR (CDCl_3_, 150 MHz) δ 177.0 (C-2), 174.9 (C-4), 82.6 (C-6), 78.5 (C-6a), 49.1 (C-3a), 38.5 (C-3), 28.7 (C-7), 27.6 (C-8), 22.6 (C-9), 17.3 (Me), 14.0 (C-10). ${\left[\alpha \right]}_{D}^{20}$ − 24.0 (c 0.10, MeOH).

#### 4.3.3. Kojic Acid (**3**)

^1^H NMR (CD_3_OD, 400 MHz) δ 7.95 (1H, s, H-6), 6.50 (1H, s, H-3), 4.41 (2H, s, H_2_-7); ^13^C NMR (CD_3_OD, 100 MHz) δ 175.5 (C-4), 169.0 (C-2), 146.0 (C-5), 139.6 (C-6), 109.4 (C-3), 59.8 (C-7).

#### 4.3.4. Citreoisocoumarin (**4**)

^1^H NMR (CD_3_OD, 600 MHz) δ 6.38 (1H, s, H-4), 6.315 (1H, d, *J* = 2.2 Hz, H-7), 6.311 (1H, d, *J* = 3.0 Hz, H-5), 4.47 (1H, m, H-10), 2.70 (2H, d, *J* = 6.4 Hz, H_2_-11), 2.67 (1H, dd, *J* = 4.9, 15.5 Hz, H_2_-9a), 2.62 (1H, dd, *J* = 8.0, 14.5 Hz, H_2_-9b), 2.18 (3H, s, H_3_-13); ^13^C NMR (CD_3_OD, 150 MHz) δ 210.0 (C-12), 167.9 (C-8), 167.7 (C-6), 165.0 (C-1), 155.7 (C-3), 141.3 (C-4a), 107.5 (C-4), 104.0 (C-5), 102.9 (C-7), 99.9 (C-8a), 66.5 (C-10), 51.2 (C-11), 42.1 (C-9), 30.8 (C-13).

#### 4.3.5. Astellolide A (**5**)

^1^H NMR (CDCl_3_, 600 MHz) δ 8.02 (2H, dt, *J* = 1.3, 7.1 Hz, H-3′, H-7′), 7.60 (1H, tt, *J* = 1.1, 7.4 Hz, H-5′), 7.51 (2H, td, *J* = 1.5, 7.6 Hz, H-4′, H-6′), 5.95 (1H, dt, *J* = 5.2 Hz, H-6), 5.01 (1H, dt, *J* = 2.8, 17.3 Hz, H_2_-11a), 4.85 (1H, dq, *J* = 1.9, 17.3 Hz, H_2_-11b), 4.97 (1H, d, *J* = 10.8 Hz, H_2_-15a), 4.89 (1H, d, *J* = 10.8 Hz, H_2_-15b), 4.42 (1H, d, *J* = 11.4 Hz, H_2_-14a), 4.40 (1H, d, *J* = 11.4 Hz, H_2_-14b), 2.78 (1H, dd, *J* = 3.1, 19.7 Hz, H_2_-7a), 2.72 (1H, m, H_2_-7b), 2.29 (1H, dt, *J* = 12.8 Hz, H_2_-1a), 1.38 (1H, td, *J* = 3.0, 13.3 Hz, H_2_-1b), 2.13 (3H, s, OAc), 1.93 (1H, dt, *J* = 15.1 Hz, H_2_-3a), 1.26 (1H, td, *J* = 4.0, 13.8 Hz, H_2_-3b), 1.88 (3H, s, OAc), 1.81 (1H, s, H-5), 1.79 (1H, m, H_2_-2a), 1.66 (1H, m, H_2_-2b), 1.16 (3H, s, H_3_-13); ^13^C NMR (CDCl_3_, 150 MHz) δ 173.4 (C-12), 170.9 (OAc), 170.4 (OAc), 166.3 (C-1′), 165.7 (C-9), 133.8 (C-5′), 129.8 (C-3′, C-7′), 129.6 (C-2′), 129.0 (C-4′, C-6′), 123.1 (C-8), 71.5 (C-11), 67.2 (C-14), 66.7 (C-6), 66.2 (C-15), 54.4 (C-5), 40.7 (C-10), 38.0 (C-4), 37.3 (C-3), 32.0 (C-1), 29.3 (C-7), 28.0 (C-13), 21.2 (OAc), 20.8 (OAc), 18.1 (C-2); (+)-HRESIMS *m*/*z* 488.2275 [M + NH_4_]^+^ (calcd for C_26_H_34_NO_8_^+^, 488.2279).

#### 4.3.6. Astellolide B (**6**)

^1^H NMR (CDCl_3_, 400 MHz) δ 7.90 (2H, d, *J* = 8.6 Hz, H-3′, H-7′), 6.86 (2H, d, *J* = 8.7 Hz, H-4′, H-6′), 5.91 (1H, d, *J* = 5.3 Hz, H-6), 5.00 (1H, d, *J* = 17.3 Hz, H_2_-11a), 4.85 (1H, d, *J* = 17.6 Hz, H_2_-11b), 4.95 (1H, d, *J* = 11.0 Hz, H_2_-15a), 4.87 (1H, d, *J* = 11.1 Hz, H_2_-15b), 4.39 (1H, d, *J* = 11.3 Hz, H_2_-14a), 4.00 (1H, d, *J* = 11.3 Hz, H_2_-14b), 2.76 (1H, d, *J* = 19.7 Hz, H_2_-7a), 2.70 (1H, d, *J* = 19.6 Hz, H_2_-7b), 2.28 (1H, d, *J* = 13.1 Hz, H_2_-1a), 1.37 (1H, dt, *J* = 3.2, 13.0 Hz, H_2_-1b), 2.13 (3H, s, OAc), 1.93 (1H, d, *J* = 12.9 Hz, H_2_-3a), 1.26 (1H, dt, *J* = 3.7, 13.8 Hz, H_2_-3b), 1.91 (3H, s, OAc), 1.79 (1H, s, H-5), 1.78 (1H, m, H_2_-2a), 1.66 (1H, m, H_2_-2b), 1.16 (3H, s, H_3_-13); ^13^C NMR (CDCl_3_, 100 MHz) δ 173.6 (C-12), 171.4 (OAc), 170.6 (OAc), 166.0 (C-1′), 165.8 (C-9), 161.0 (C-5′), 132.2 (C-3′, C-7′), 123.1 (C-8), 121.7 (C-2′), 115.9 (C-4′, C-6′), 71.5 (C-11), 67.4 (C-14), 66.4 (C-15), 66.3 (C-6), 54.6 (C-5), 40.8 (C-10), 38.0 (C-4), 37.3 (C-3), 32.1 (C-1), 29.4 (C-7), 28.0 (C-13), 21.1 (OAc), 20.8 (OAc), 18.1 (C-2); (+)-HRESIMS *m/z* 504.2224 [M + NH_4_]^+^ (calcd for C_26_H_34_NO_9_^+^, 504.2228).

#### 4.3.7. Astellolide G (**7**)

^1^H NMR (CDCl_3_, 400 MHz) δ 7.81 (2H, d, *J* = 8.5 Hz, H-3′, H-7′), 6.81 (2H, d, *J* = 8.4 Hz, H-4′, H-6′), 5.91 (1H, d, *J* = 5.1 Hz, H-6), 5.16 (1H, d, *J* = 17.6 Hz, H_2_-11a), 4.79 (1H, d, *J* = 18.4 Hz, H_2_-11b), 4.43 (1H, d, *J* = 10.5 Hz, H_2_-15a), 4.33 (1H, d, *J* = 10.1 Hz, H_2_-15b), 4.45 (1H, d, *J* = 11.3 Hz, H_2_-14a), 3.87 (1H, d, *J* = 11.1 Hz, H_2_-14b), 2.71 (2H, m, H_2_-7), 2.42 (1H, d, J = 12.4 Hz, H_2_-1a), 1.29 (1H, m, H_2_-1b), 1.93 (3H, s, OAc), 1.90 (1H, d, *J* = 14.0 Hz, H_2_-3a), 1.18 (1H, d, *J* = 13.3 Hz, H_2_-3b), 1.76 (1H, s, H-5), 1.72 (1H, d, *J* = 13.2 Hz, H_2_-2a), 1.62 (1H, d, *J* = 14.3 Hz, H_2_-2b), 1.17 (3H, s, H_3_-13); ^13^C NMR (CDCl_3_, 100 MHz) δ 174.3 (C-12), 172.0 (OAc), 167.7 (C-9), 166.0 (C-1′), 161.0 (C-5′), 132.0 (C-3′, C-7′), 122.0 (C-8), 122.0 (C-2′), 115.8 (C-4′, C-6′), 72.3 (C-11), 67.5 (C-14), 64.3 (C-15), 66.7 (C-6), 54.5 (C-5), 43.2 (C-10), 38.1 (C-4), 37.0 (C-3), 32.0 (C-1), 29.4 (C-7), 27.9 (C-13), 21.0 (OAc), 18.1 (C-2); (+)-HRESIMS *m*/*z* 462.2119 [M + NH^4^]^+^ (calcd for C_24_H_32_NO_8_^+^, 462.2122).

#### 4.3.8. *Cyclo*-N-Methylphenylalanyltryptophenyl (**8**)

^1^H NMR (CD_3_OD, 400 MHz) δ 7.54 (1H, d, *J* = 7.9 Hz, H-6), 7.35 (1H, d, *J* = 8.1 Hz, H-3), 7.26 (2H, t, *J* = 6.9 Hz, H-21, H-23), 7.21 (1H, t, *J* = 7.1 Hz, H-22), 7.15 (1H, t, *J* = 7.2 Hz, H-4), 7.06 (1H, t, *J* = 7.3 Hz, H-5), 6.97 (1H, s, H-9), 6.81 (2H, d, *J* = 7.0 Hz, H-20, H-24), 4.14 (1H, dd, *J* = 3.8, 7.0 Hz, H-11), 4.07 (1H, dd, *J* = 4.6, 5.8 Hz, H-15), 2.97 (1H, dd, *J* = 3.8, 14.5 Hz, H_2_-10a), 2.25 (1H, dd, *J* = 7.2, 14.4 Hz, H_2_-10b), 2.75 (1H, dd, *J* = 4.4, 14.2 Hz, H_2_-18a), 2.06 (1H, dd, *J* = 6.3, 14.1 Hz, H_2_-18b), 2.69 (3H, s, H_3_-14); ^13^C NMR (CD_3_OD, 100 MHz) δ 169.0 (C-16), 168.6 (C-12), 138.3 (C-2), 138.1 (C-19), 131.0 (C-20, C-24), 129.9 (C-21, C-23), 128.8 (C-7), 128.3 (C-22), 125.7 (C-9), 122.8 (C-4), 120.3 (C-5), 119.9 (C-6), 112.7 (C-3), 110.1 (C-8), 65.1 (C-15), 57.4 (C-11), 39.6 (C-18), 34.5 (C-14), 31.8 (C-10); (+)-HRESIMS *m/z* 348.1711 [M + H]^+^ (calcd for C_21_H_22_N_3_O_2_^+^, 348.1707).

#### 4.3.9. (−)-Ditryptophenaline (**9**)

^1^H NMR (CDCl_3_, 400 MHz) δ 7.57 (2H, t, *J* = 7.0 Hz, H-22, H-22), 7.51 (1H, t, *J* = 7.2 Hz, H-21), 7.14 (2H, d, *J* = 7.2 Hz, H-19, H-23), 7.08 (1H, t, *J* = 7.6 Hz, H-6), 6.97 (1H, d, *J* = 7.5 Hz, H-5), 6.71 (1H, t, *J* = 7.5 Hz, H-7), 6.56 (1H, d, *J* = 7.8 Hz, H-8), 4.81 (1H, s, H-2), 4.25 (1H, m, H-15), 3.68 (1H, dd, *J* = 4.6, 11.8 Hz, H-11), 3.54 (1H, dd, *J* = 3.0, 14.2 Hz, H_2_-17a), 3.27 (1H, dd, *J* = 4.3, 14.2 Hz, H_2_-17b), 3.02 (3H, s, H_3_-24), 2.04 (1H, dd, *J* = 4.8, 12.3 Hz, H_2_-12a), 1.60 (1H, t, *J* = 12.1 Hz, H_2_-12b); ^13^C NMR (CDCl_3_, 100 MHz) δ 165.6 (C-13), 164.1 (C-16), 150.3 (C-9), 134.7 (C-18), 129.8 (C-6), 129.55 (C-20, C-22), 129.49 (C-19, C-23), 128.1 (C-21), 126.6 (C-4), 125.9 (C-5), 119.1 (C-7), 109.8 (C-8), 78.8 (C-2), 63.3 (C-15), 59.1 (C-3), 58.7 (C-11), 36.4 (C-17), 36.2 (C-12), 32.7 (C-24); (+)-HRESIMS *m/z* 693.3187 [M + H]^+^ (calcd for C_42_H_41_N_6_O_4_^+^, 693.3184).

### 4.4. X-ray Crystallographic Analysis of Compound **2**

The crystal of **2** was obtained by recrystallization from MeOH, and the X-ray crystallographic data were collected on a Bruker D8 venture diffractometer at 296 (2) K using monochromatic Cu Kα radiation (λ = 1.54178 Å). The programs ShelXS^17^ and ShelXL-2014/7 were used for structure solution and refinement.

C_11_H_16_O_4_, monoclinic, a = 6.6129 (8) Å, b = 6.6141 (8) Å, c = 13.4692 (16) Å, α = 80.258 (8)°, β = 80.195 (7)°, γ = 81.731 (8)°, *V* = 568.12 (12) Å^3^, Z = 2, *D_calcd_* = 1.282 g/cm^3^, μ (Cu Kα) = 0.810 mm^−1^, F (000) = 235. The flack parameter was −0.1 (4). Details of crystallographic data for **2** have been deposited with the Cambridge Crystallographic Data Centre (CCDC) as CCDC number 221329.

### 4.5. Germination-Inhibition Assay

The anti-germination activity of crude extracts of *Aspergillus* sp. MJ01 was carried out as follows: the crude extract was dissolved in MeOH and prepared into the solution with a concentration of 50 μg/μL. 1 μL, 5 μL and 25 μL of each crude extract were added to the 6 mm filter paper. Upon evaporation of MeOH, the 6 mm filter paper filled with different volumes of crude extract was placed on a 8 cm filter paper in the 9 cm petri dish, respectively. Afterwards, the 8 cm filter paper was moistened with 1.6 mL of distilled water and then 15 seeds of *A. thaliana* were arranged in each 6 mm filter paper and allowed to grow in a growth chamber (23 ± 2 °C, 75% relative humidity, with a photoperiod of 16:8 light-dark cycle) for 5 days. 

The anti-germination activity of compounds **1**–**9** was evaluated as follows: compounds **1**–**9** were dissolved in MeOH and prepared into the solution with a concentration of 1 μg/μL. 10 μL, 20 μL and 40 μL of each purified compound were added to the 30 mm filter paper displayed in the 6 wall plate. Upon evaporation of methanol, the filter paper was moistened with 0.2 mL of distilled water and then 25 seeds of *A. thaliana* were arranged in each 30 mm filter paper and allowed to grow in a growth chamber (23 ± 2 °C, 75% relative humidity, with a photoperiod of 16:8 light-dark cycle) for 5 days. 

The inhibitory effects were observed after 72 h. MICs of active compounds were the minimum concentration of these compounds that can completely inhibit the seed germination of *A. thaliana*. Treatments were arranged in a completely randomized design with three replications. Control petri dishes contained only methanol and distilled water. The standard for seed germination of *A. thaliana* was that the radicle protruded 2 mm from the testa [39]. Germination inhibition indice was calculated as *I*_G_% = [1 − (T/C)] × 100, where T and C are either the number of seeds germinated in the treatment and the control, respectively. The results of treated and control experiments were analyzed by Student’s *t* test (*p* < 0.05).

## 5. Conclusions

From the fermentation extracts of *Aspergillus* sp. MJ01, nine compounds were isolated and identified; aspertamarinoic acid (**1**) is a previous undescribed furanone. For the first time, we confirmed the phytotoxic activities of aspertamarinoic acid (**1**) and astellolide A (**5**) against the seed germination of *A. thaliana*, which enriched the structural diversity of phytotoxic natural products. Further, the mechanism of these phytotoxic compounds will be disclosed using biochemical and transcriptomic methods.

## Figures and Tables

**Figure 2:**
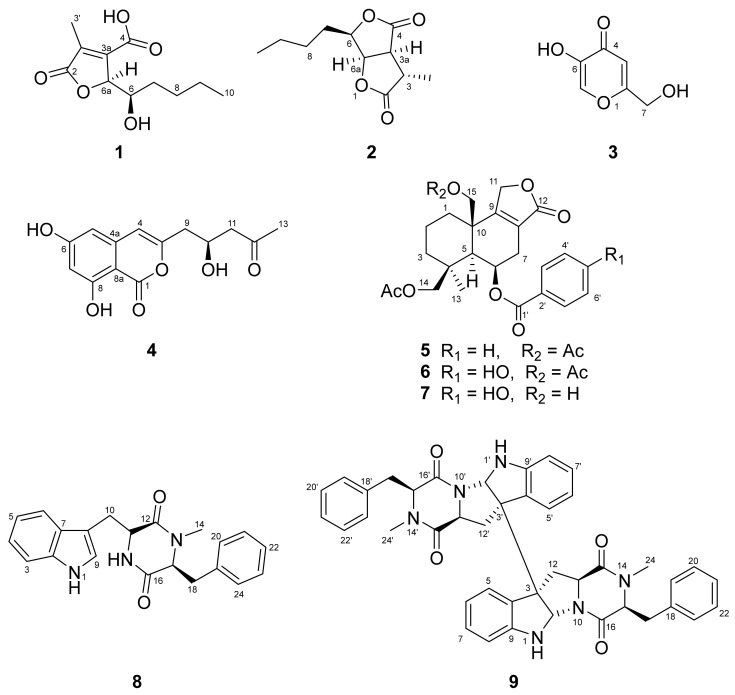
Structures of compounds **1**–**9** isolated from *Aspergillus* sp.

**Table 1:** The MIC (μg/mL) of purified compounds against the seed germination of *A. thaliana*.

Compounds	1	2	3	4	5	8
MIC (μg/mL)	200	800	400	800	200	400

## Data Availability

Not applicable.

## Supplementary Materials

The following supporting information can be downloaded at: https://www.mdpi.com/article/10.3390/molecules27227710/s1, Figures S1–S25: The 1D NMR spectra of compounds **1**–**9,** 2D NMR spectra of **1** and the morphology of strain MJ01.
