# Supplementary material for: Phytotoxic Metabolites Isolated from Aspergillus sp., an Endophytic Fungus of Crassula arborescens
Source: Molecules. 2022 Nov 9;27(22):7710. doi: 10.3390/molecules27227710 (PMC9699134; doi:10.3390/molecules27227710)
Supplement: Supplementary file 1 [file molecules-27-07710-s001.zip › molecules-2009085-supplementary.pdf]

Supporting Information

Contents

Figure S1a. Experimental ECD spectrum of **1**

Figure S1b. Experimental ECD spectrum and calculated ECD data of aspertamarinolide A

Figure S2. HRESIMS spectrum of **1**

Figure S3. ^{1}H NMR spectrum (600 MHz, CD_3OD) of **1**

Figure S4. ^{13}C NMR spectrum (150 MHz, CD_3OD) of **1**

Figure S5. HSQC spectrum of **1**

Figure S6. HMBC spectrum of **1**

Figure S7. ^{1}H-^{1}H COSY spectrum of **1**

Figure S8. NOESY spectrum of **1**

Figure S9. ^{1}H NMR spectrum (600 MHz, CD_3OD) of **2**

Figure S10. ^{13}C NMR spectrum (150 MHz, CD_3OD) of **2**

Figure S11. ^{1}H NMR spectrum (400 MHz, CD_3OD) of **3**

Figure S12. ^{13}C NMR spectrum (100 MHz, CD_3OD) of **3**

Figure S13. ^{1}H NMR spectrum (600 MHz, CD_3OD) of **4**

Figure S14. ^{13}C NMR spectrum (150 MHz, CD_3OD) of **4**

Figure S15. ^{1}H NMR spectrum (600 MHz, $CDCl_3$) of **5**

Figure S16. ^{13}C NMR spectrum (150 MHz, $CDCl_3$) of **5**

Figure S17. ^{1}H NMR spectrum (400 MHz, $CDCl_3$) of **6**

Figure S18. ^{13}C NMR spectrum (100 MHz, $CDCl_3$) of **6**

Figure S19. ^{1}H NMR spectrum (400 MHz, $CDCl_3$) of **7**

Figure S20. ^{13}C NMR spectrum (100 MHz, $CDCl_3$) of **7**

Figure S21. ^{1}H NMR spectrum (400 MHz, CD_3OD) of **8**

Figure S22. ^{13}C NMR spectrum (100 MHz, CD_3OD) of **8**

Figure S23. ^{1}H NMR spectrum (400 MHz, $CDCl_3$) of **9**

Figure S24. ^{13}C NMR spectrum (100 MHz, $CDCl_3$) of **9**

Figure S25. The colony and microscopic morphology of strain MJ01

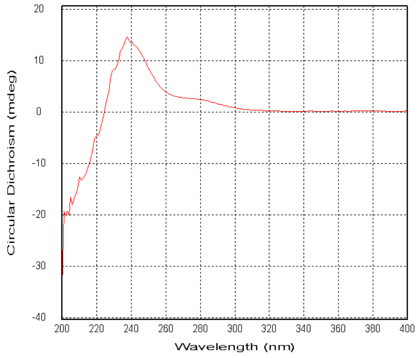

Figure S1a. Experimental ECD spectrum of **1**

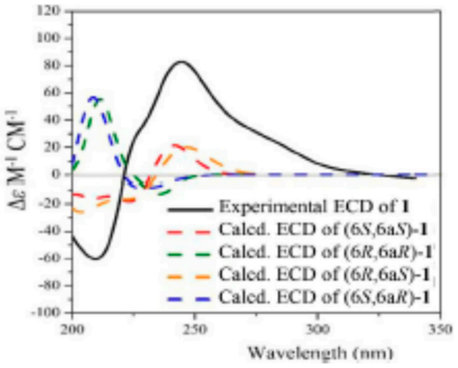

Figure S1b. Experimental ECD spectrum and calculated ECD data of aspertamarinolide A

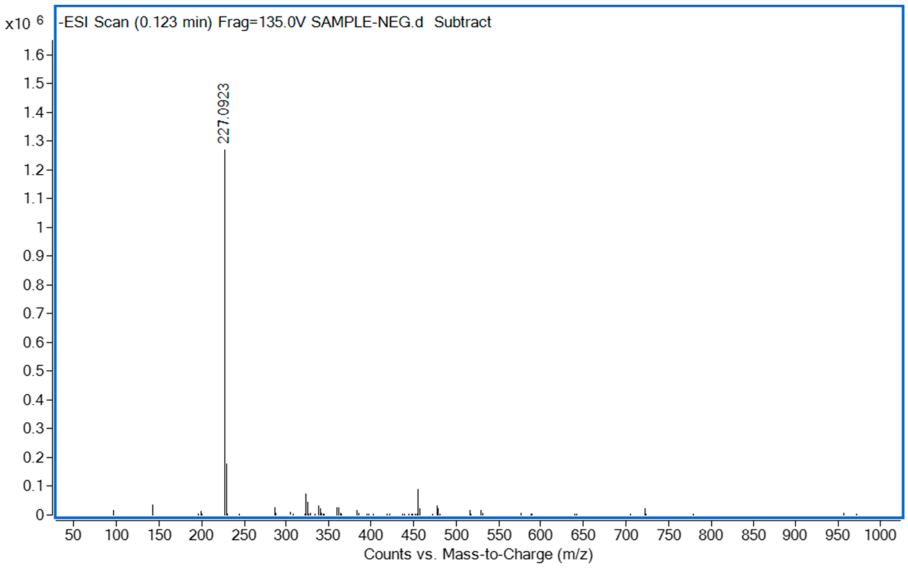

Figure S2. HRESIMS spectrum of **1**

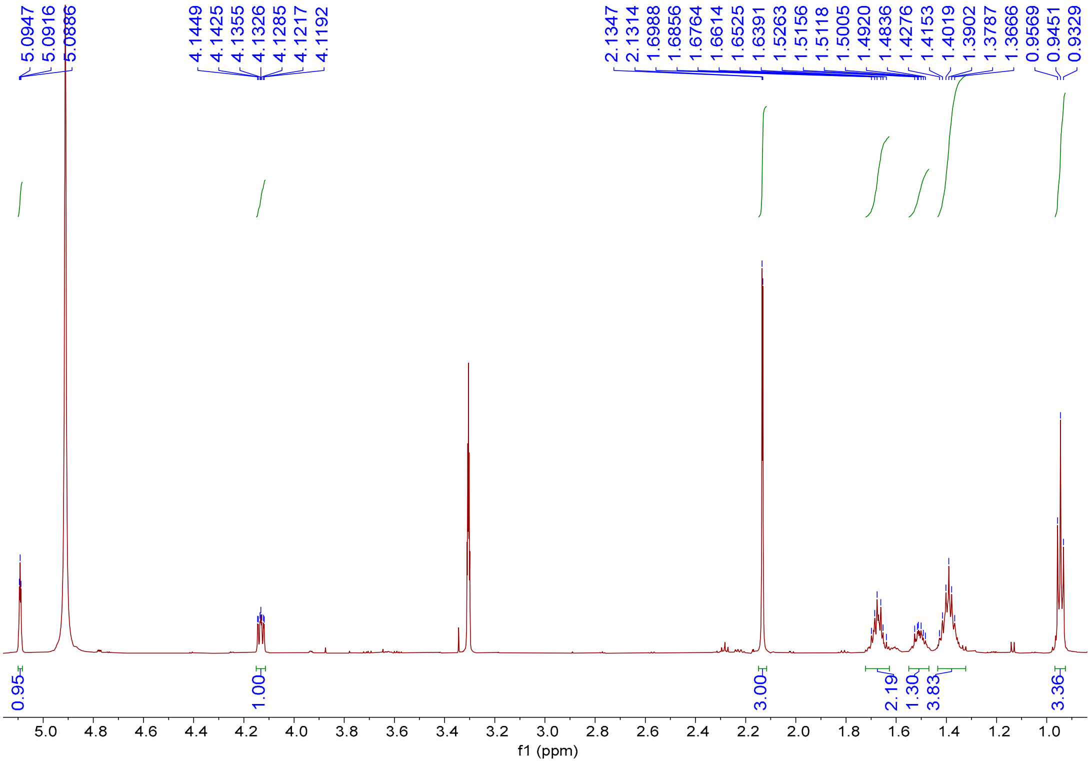

Figure S3. ^{1}H NMR spectrum (600 MHz, CD_3OD) of **1**

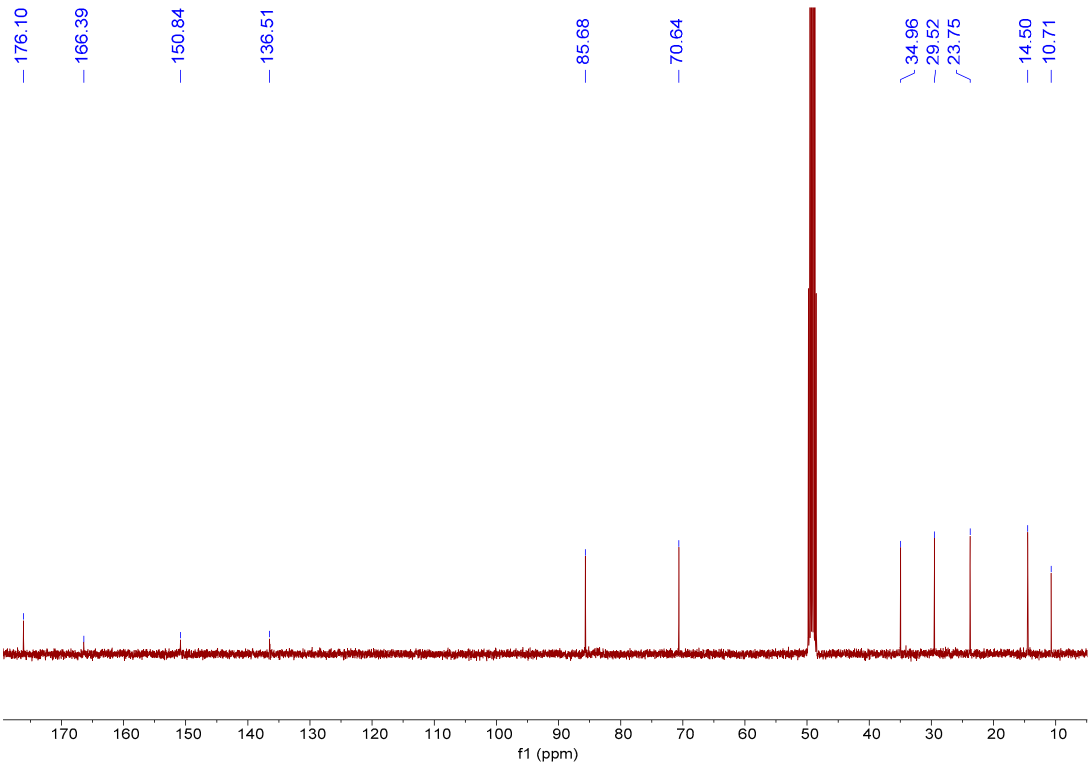

Figure S4. ^{13}C NMR spectrum (150 MHz, CD_3OD) of **1**

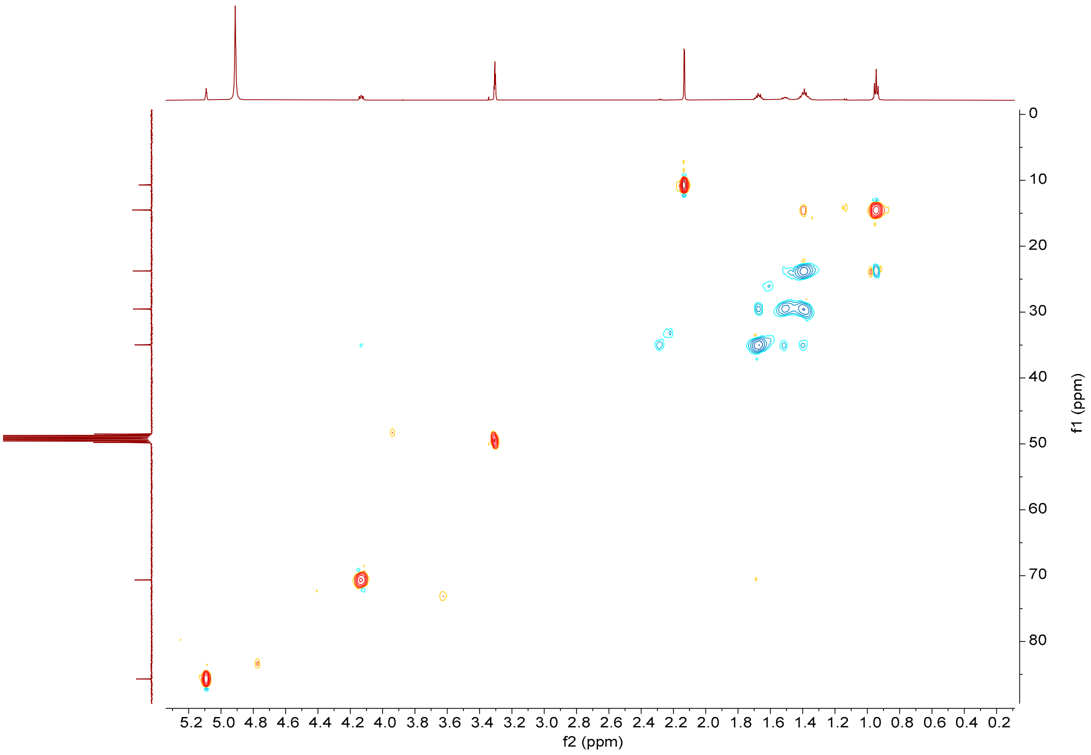

Figure S5. HSQC spectrum of **1**

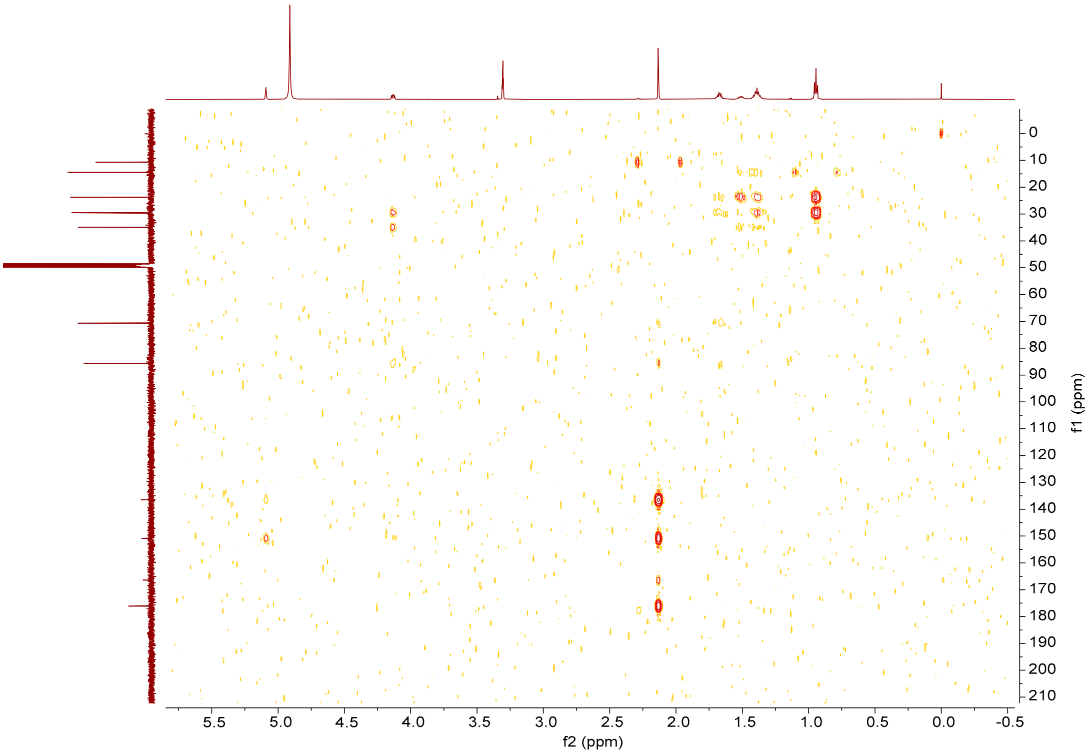

Figure S6. HMBC spectrum of **1**

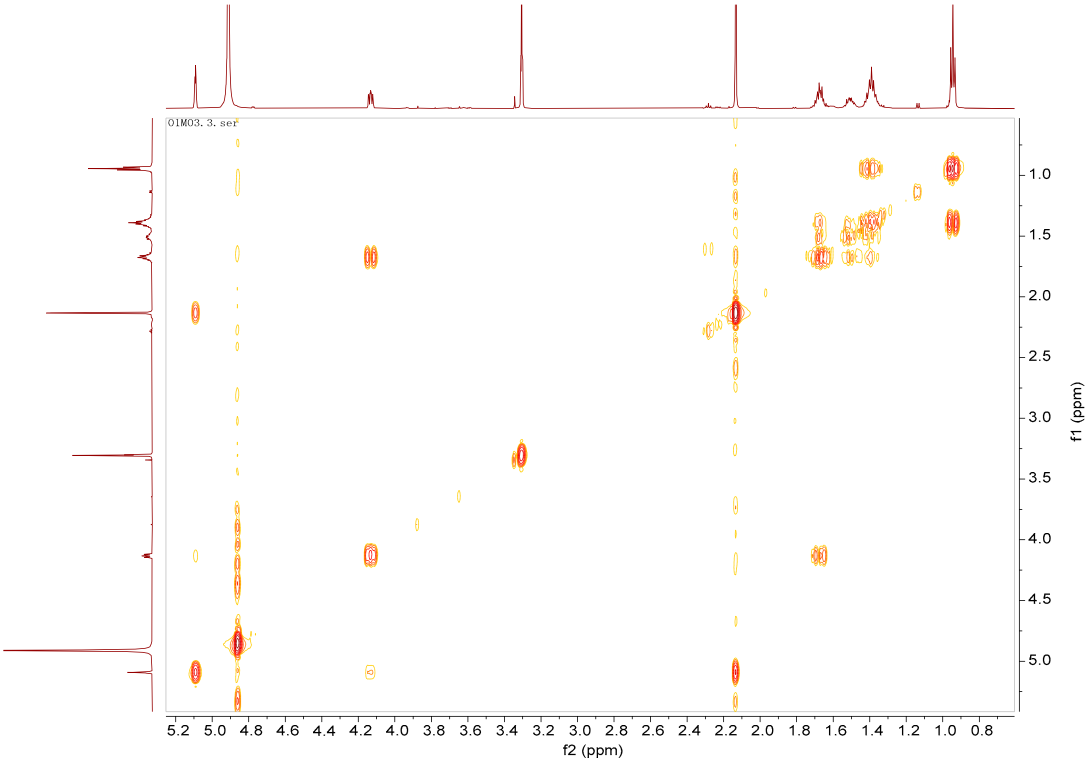

Figure S7. ^{1}H-^{1}H COSY spectrum of **1**

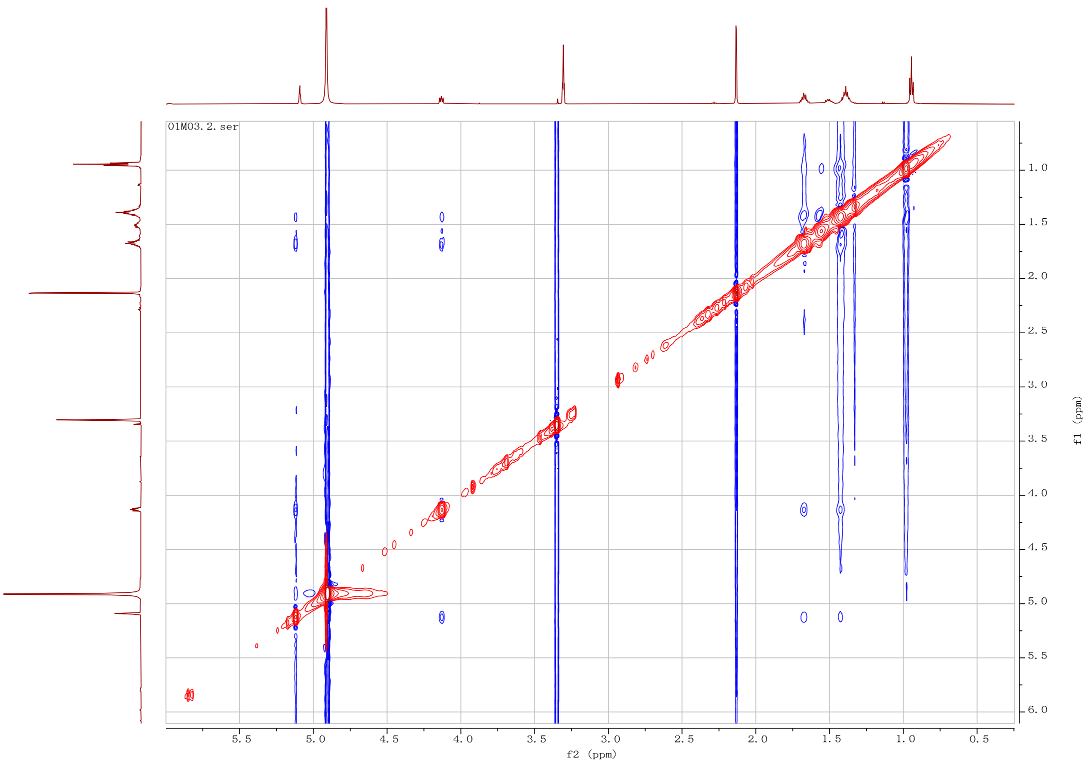

Figure S8. NOESY spectrum of **1**

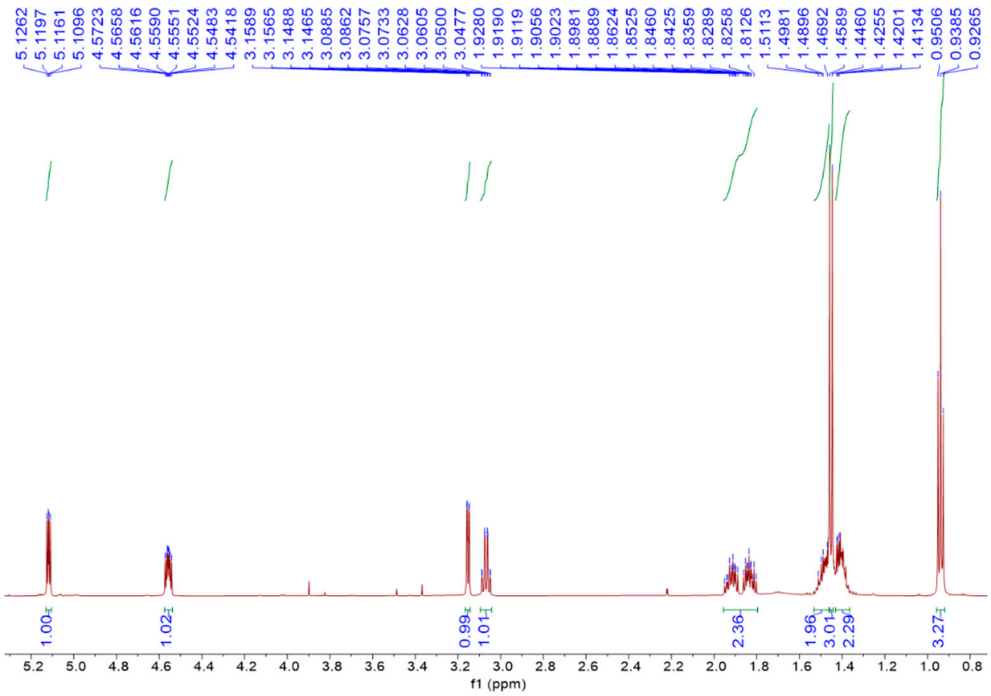

Figure S9. ^{1}H NMR spectrum (600 MHz, $CDCl_3$) of **2**

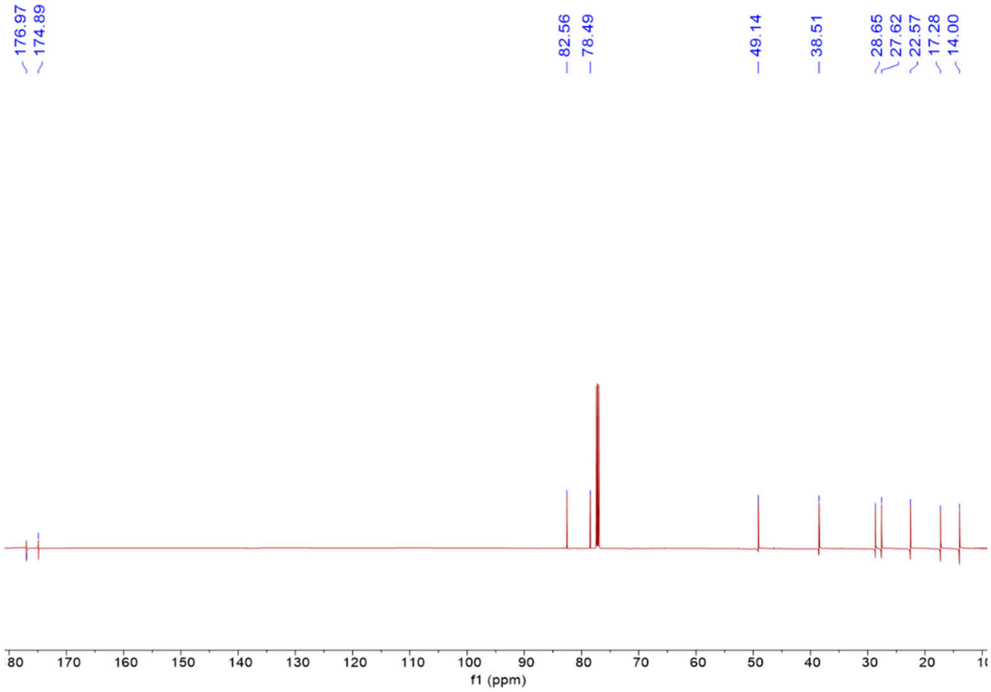

Figure S10. ^{13}C NMR spectrum (150 MHz, $CDCl_3$) of **2**

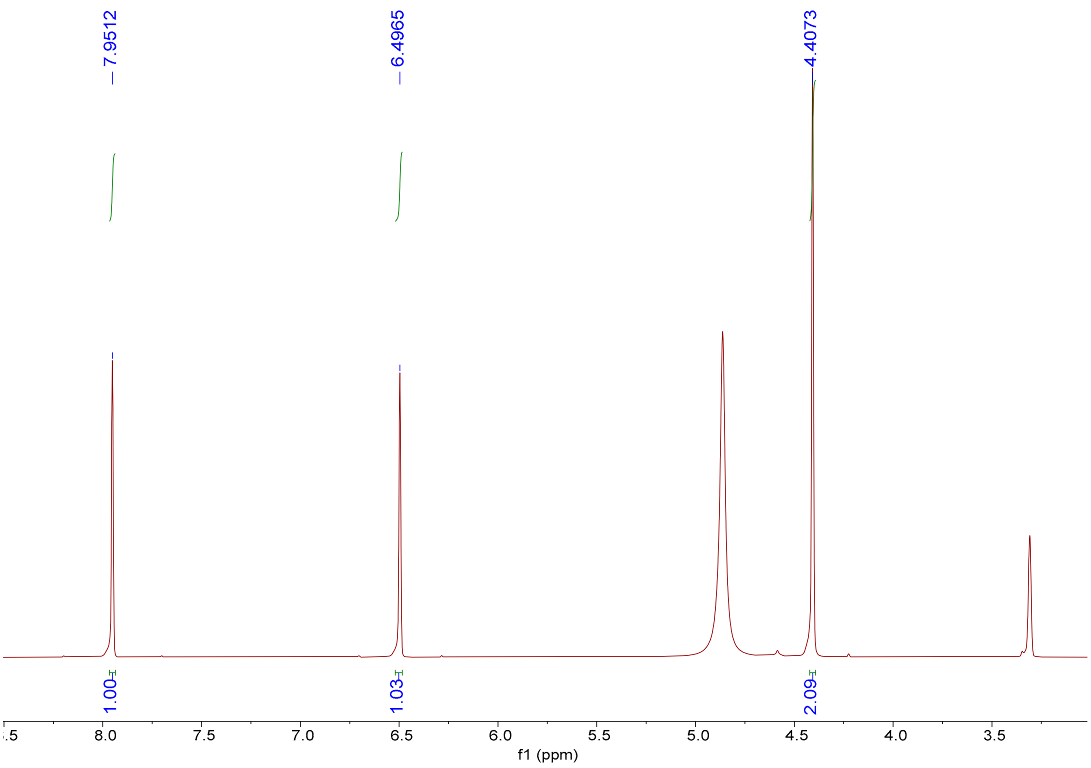

Figure S11. ^{1}H NMR spectrum (400 MHz, CD_3OD) of **3**

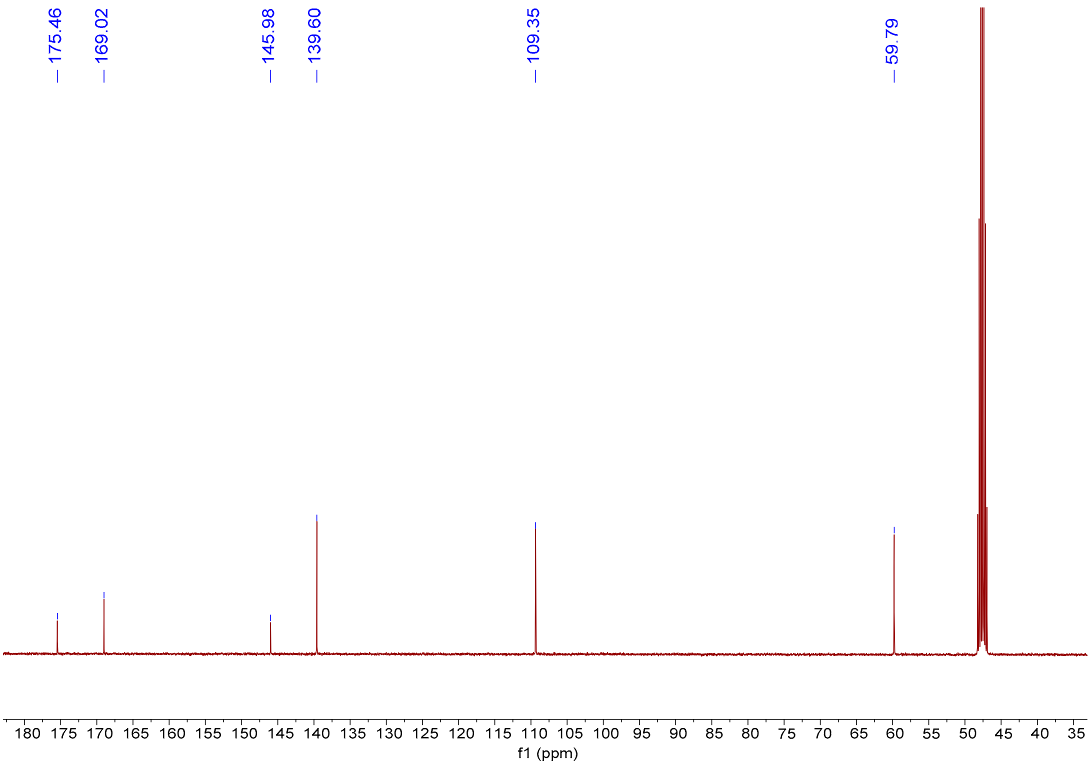

Figure S12. ^{13}C NMR spectrum (100 MHz, CD_3OD) of **3**

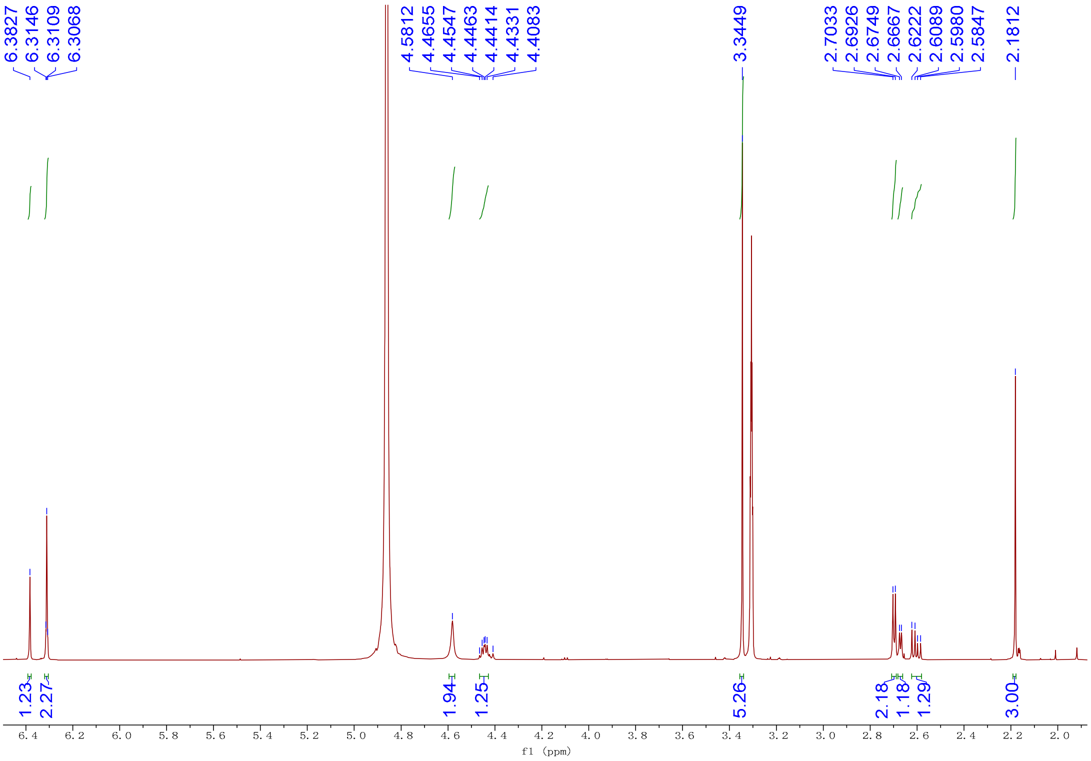

Figure S13. ^{1}H NMR spectrum (600 MHz, CD_3OD) of **4**

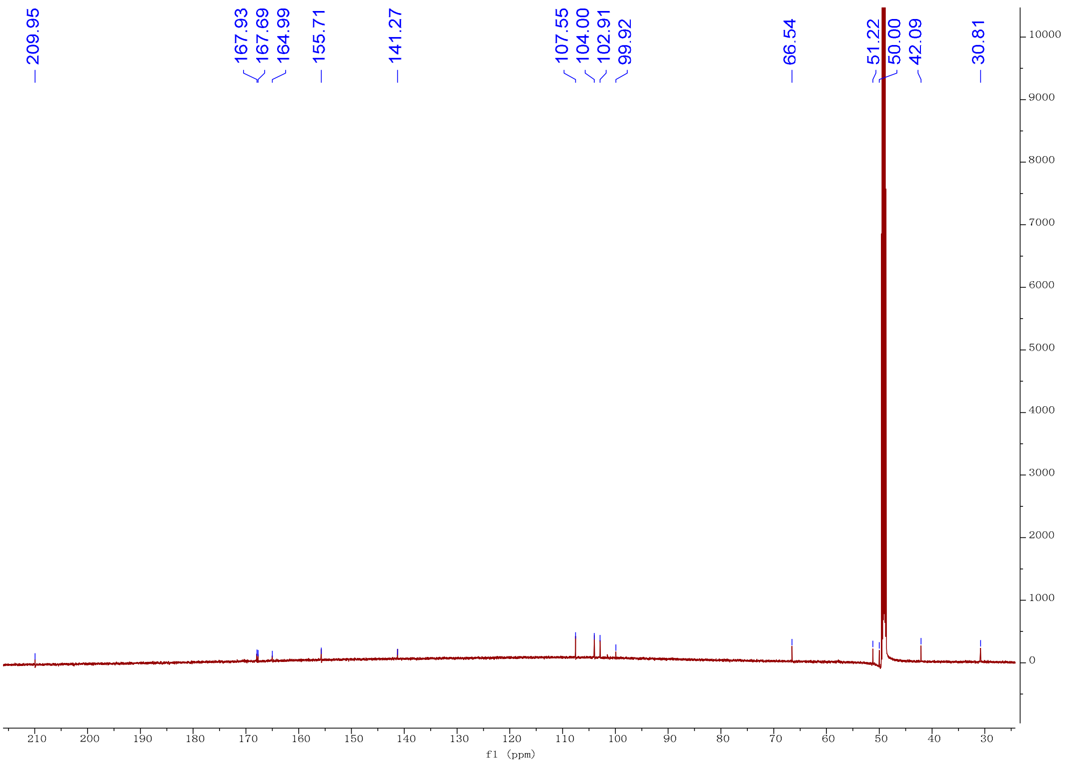

Figure S14. ^{13}C NMR spectrum (150 MHz, CD_3OD) of **4**

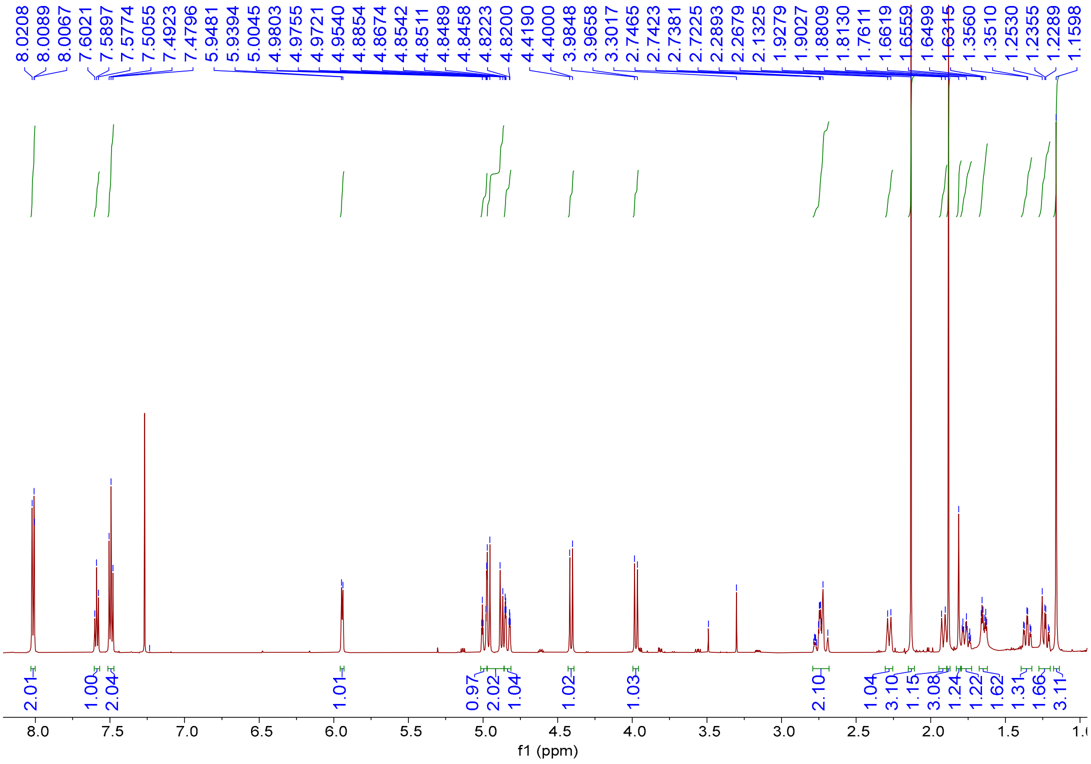

Figure S15. ^{1}H NMR spectrum (600 MHz, $CDCl_3$) of **5**

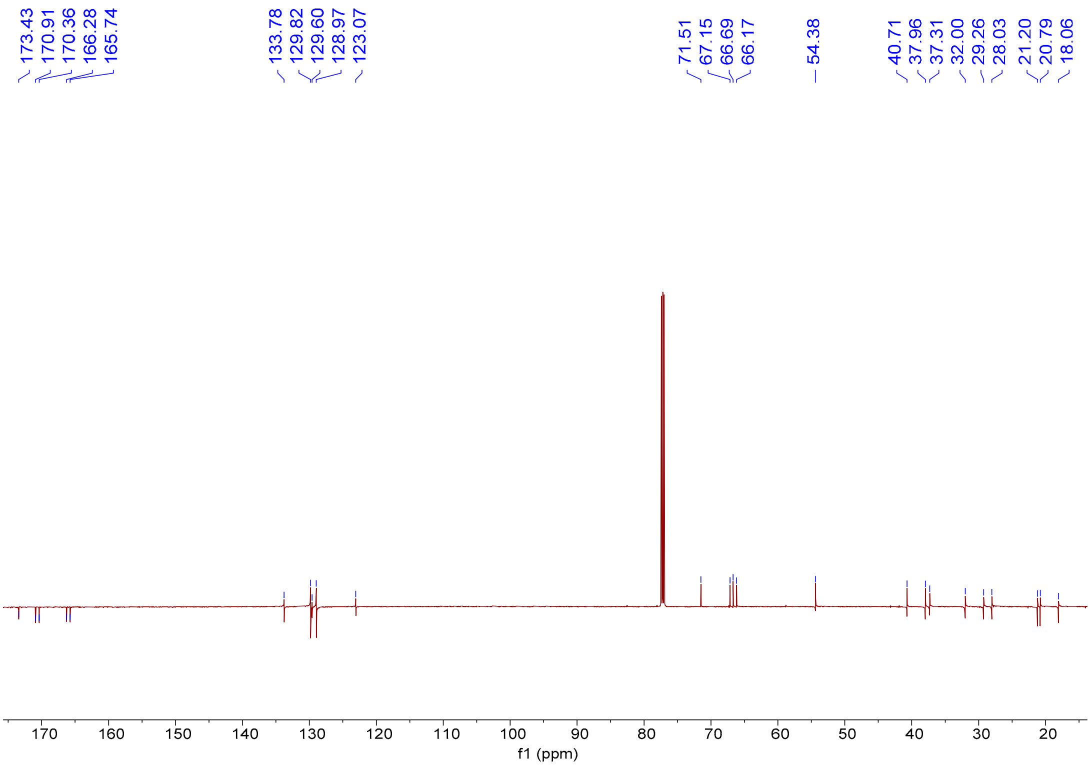

Figure S16. ^{13}C NMR spectrum (150 MHz, $CDCl_3$) of **5**

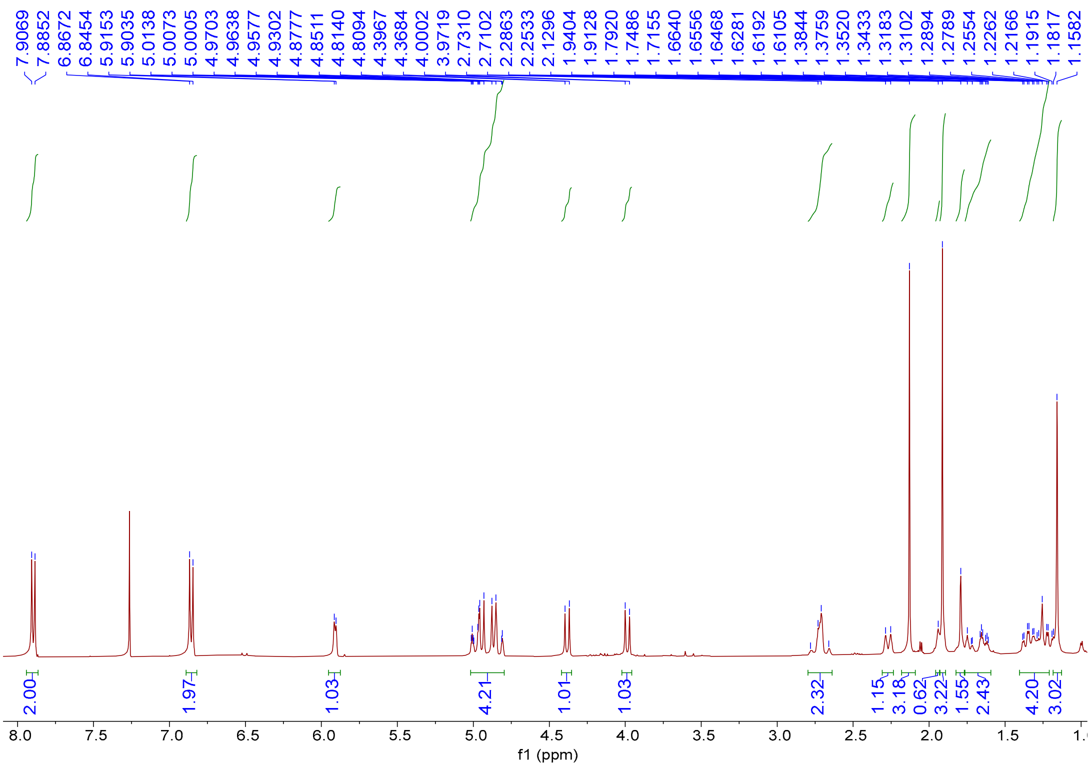

Figure S17. ^{1}H NMR spectrum (400 MHz, $CDCl_3$) of **6**

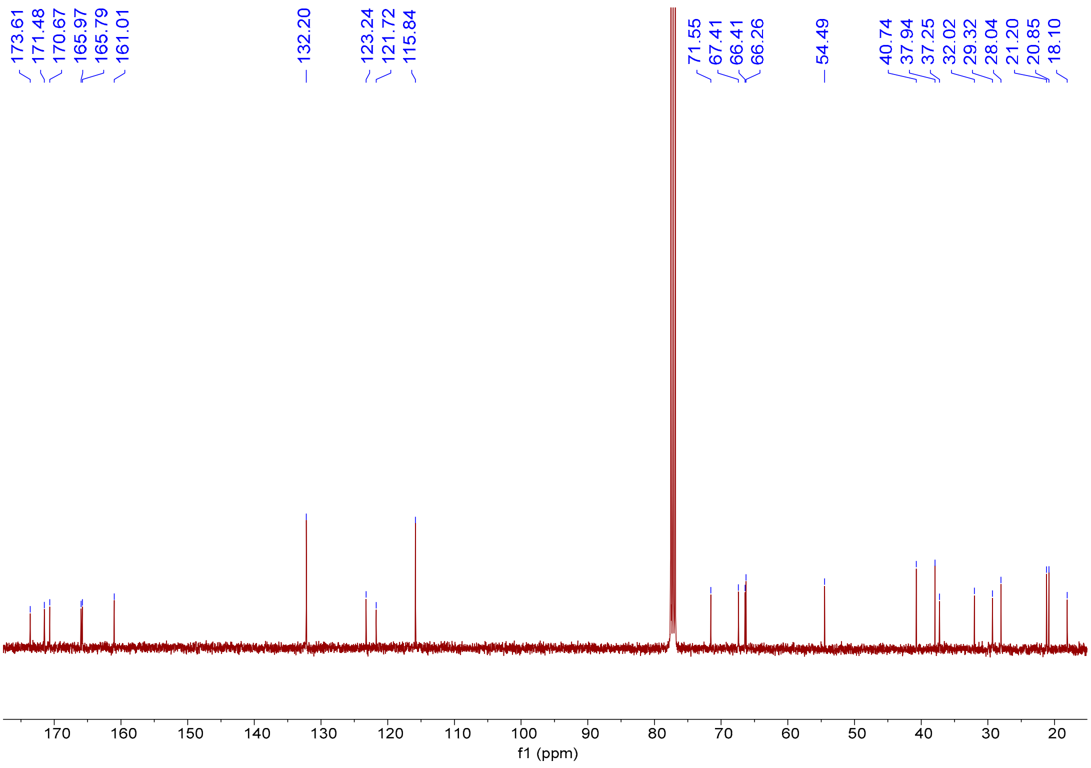

Figure S18. ^{13}C NMR spectrum (100 MHz, $CDCl_3$) of **6**

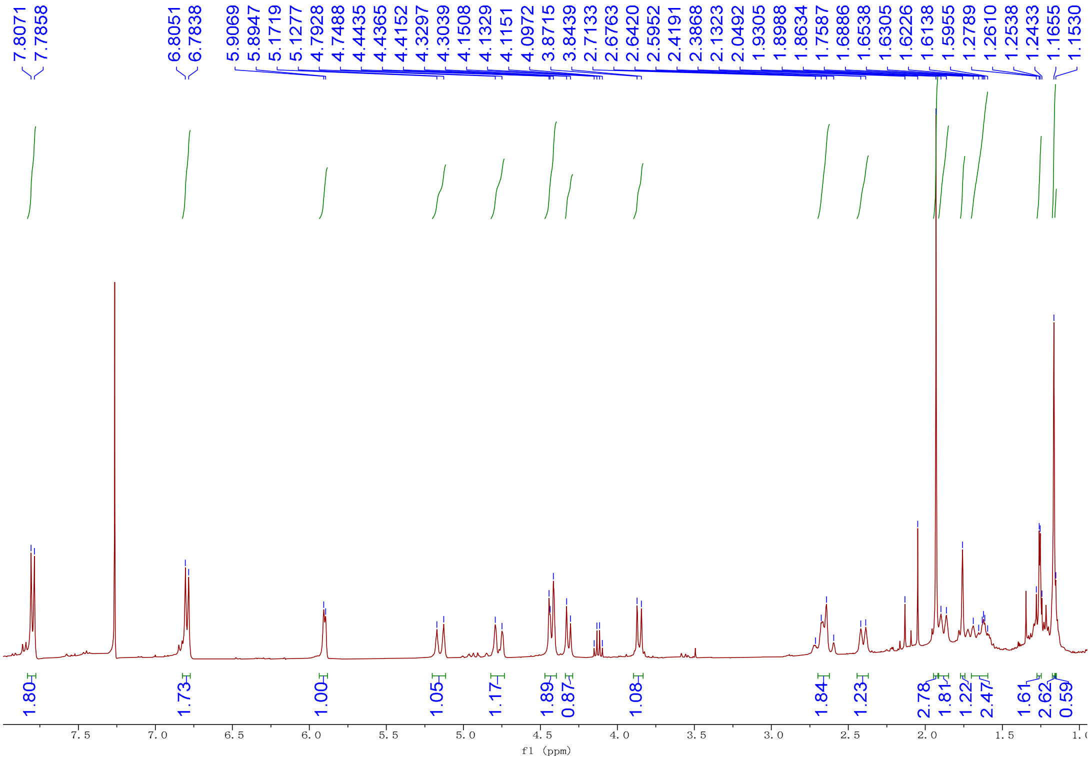

Figure S19. ^{1}H NMR spectrum (400 MHz, $CDCl_3$) of **7**

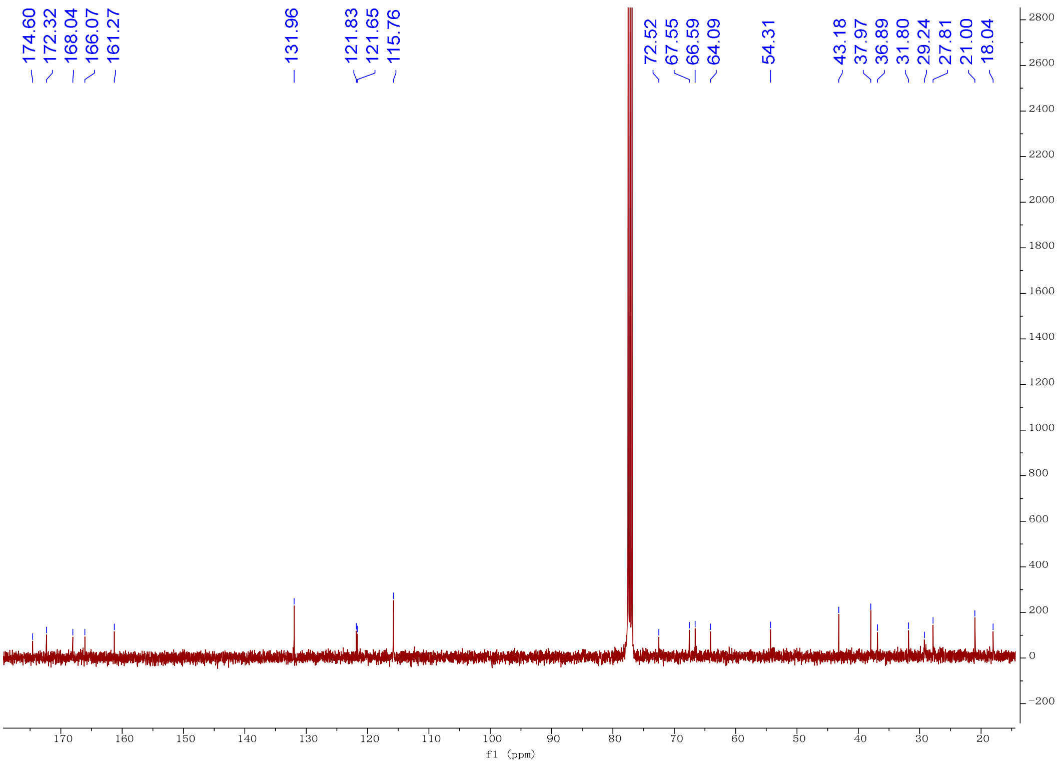

Figure S20. ^{13}C NMR spectrum (100 MHz, $CDCl_3$) of **7**

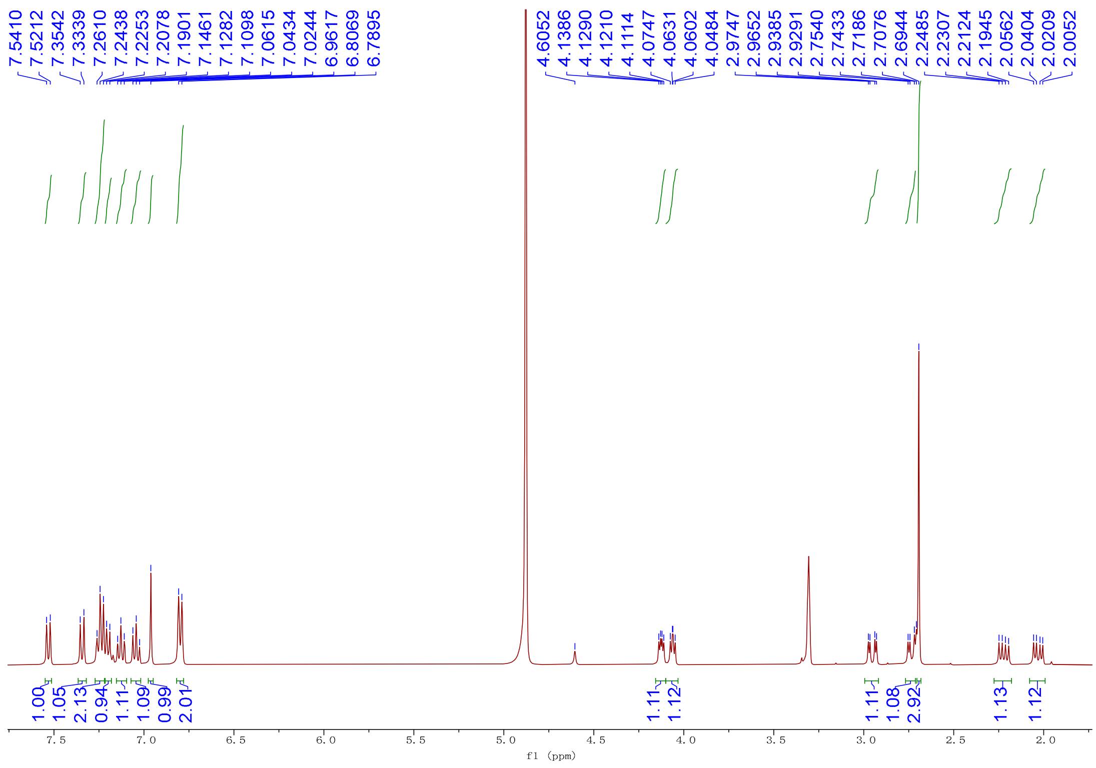

Figure S21. ^{1}H NMR spectrum (400 MHz, CD_3OD) of **8**

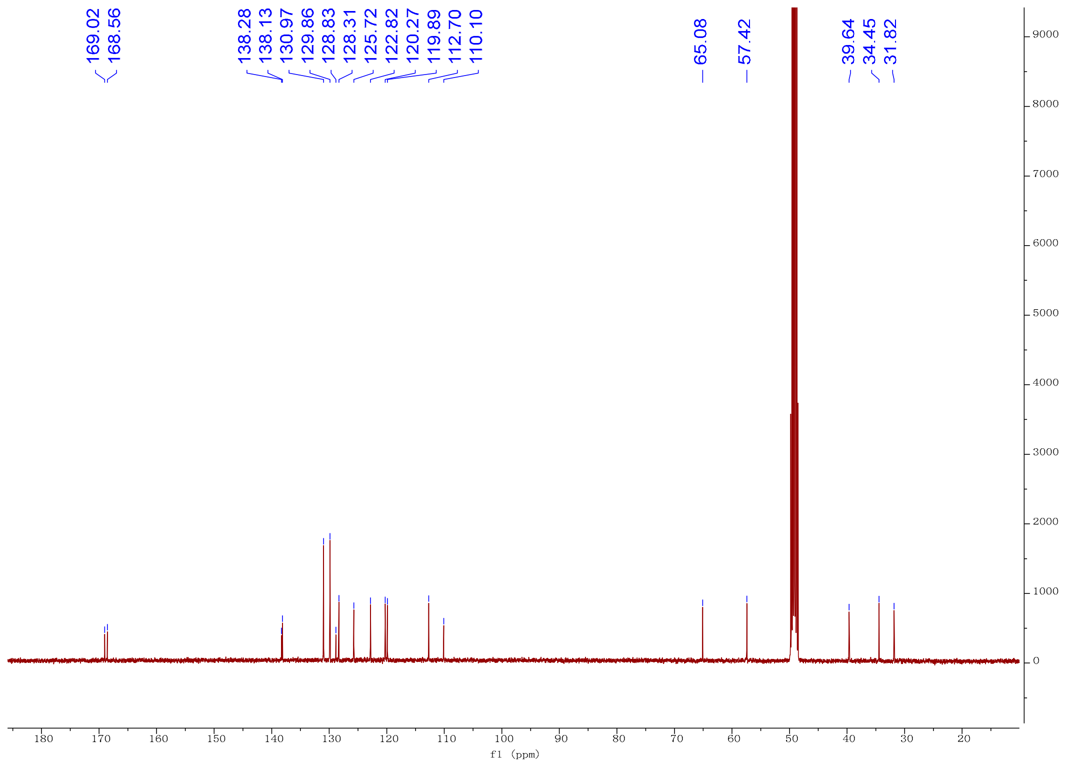

Figure S22. ^{13}C NMR spectrum (100 MHz, CD_3OD) of **8**

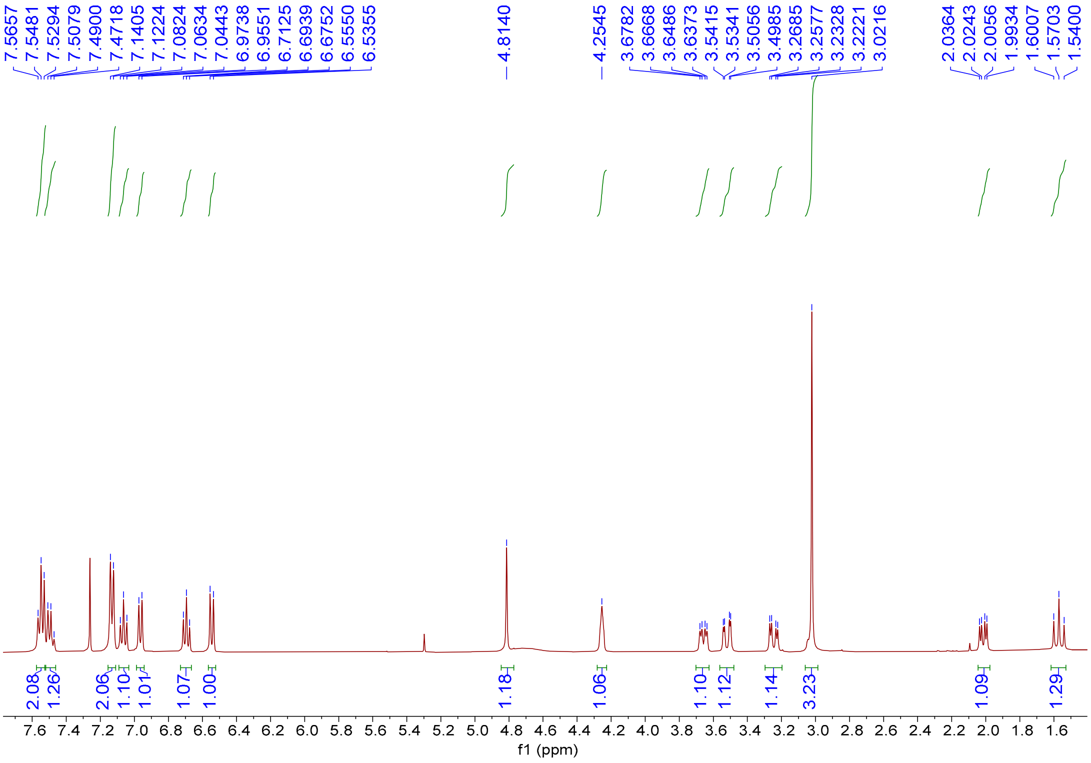

Figure S23. ^{1}H NMR spectrum (400 MHz, $CDCl_3$) of **9**

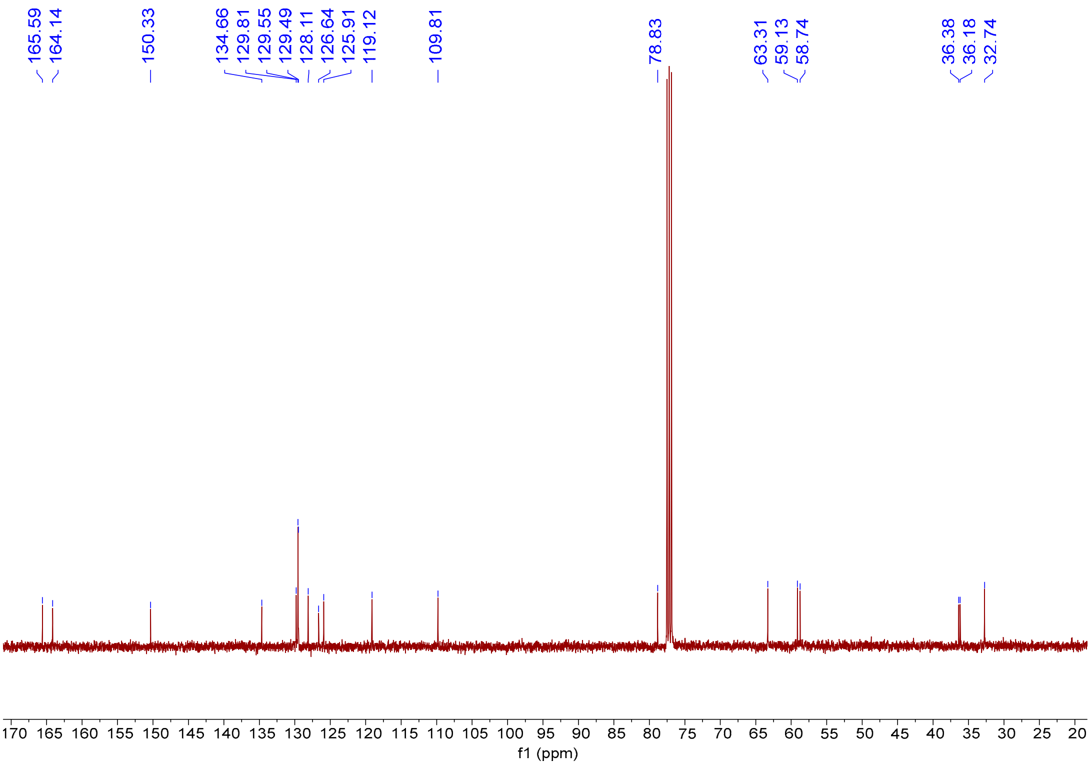

Figure S24. ^{13}C NMR spectrum (100 MHz, $CDCl_3$) of **9**

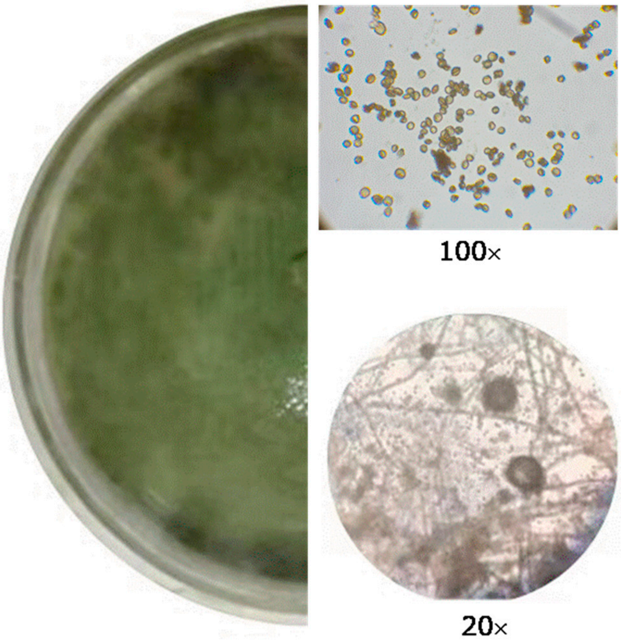

Figure S25. The colony and microscopic morphology of strain MJ01
